# Longitudinal epigenome-wide association studies of three male military cohorts reveal multiple CpG sites associated with post-traumatic stress disorder

**DOI:** 10.1186/s13148-019-0798-7

**Published:** 2020-01-13

**Authors:** Clara Snijders, Adam X. Maihofer, Andrew Ratanatharathorn, Dewleen G. Baker, Marco P. Boks, Elbert Geuze, Sonia Jain, Ronald C. Kessler, Ehsan Pishva, Victoria B. Risbrough, Murray B. Stein, Robert J. Ursano, Eric Vermetten, Christiaan H. Vinkers, PGC PTSD EWAS Consortium, Alicia K. Smith, Monica Uddin, Bart P. F. Rutten, Caroline M. Nievergelt

**Affiliations:** 1 Department of Psychiatry and Neuropsychology, School for Mental health and Neuroscience, Maastricht University, Maastricht, Limburg Netherlands; 2 Department of Psychiatry, University of California San Diego, La Jolla, CA USA; 3 Center of Excellence for Stress and Mental Health, Veterans Affairs San Diego Healthcare System, San Diego, CA USA; 4 Research Service, Veterans Affairs San Diego Healthcare System, San Diego, CA USA; 5 Department of Epidemiology, Harvard T.H. Chan School of Public Health, Boston, MA USA; 6 Psychiatry Service, Veterans Affairs San Diego Healthcare System, San Diego, CA USA; 7 Department of Psychiatry, UMC Utrecht Brain Center, Utrecht, Utrecht Netherlands; 8 Brain Research & Innovation Centre, Netherlands Ministry of Defense, Utrecht, Utrecht Netherlands; 9 Department of Family Medicine and Public Health, University of California San Diego, La Jolla, CA USA; 10 Department of Health Care Policy, Harvard Medical School, Boston, MA USA; 11 College of Medicine and Health, University of Exeter Medical School, Exeter, UK; 12 Million Veteran Program, Veterans Affairs San Diego Healthcare System, San Diego, CA USA; 13 Department of Psychiatry, Uniformed Services University, Bethesda, MD USA; 14 Arq, Psychotrauma Research Expert Group, Diemen, North Holland Netherlands; 15 Department of Psychiatry, Leiden University Medical Center, Leiden, South Holland Netherlands; 16 Military Mental Healthcare, Netherlands Ministry of Defense, Utrecht, Utrecht Netherlands; 17 Department of Psychiatry, New York University School of Medicine, New York, NY USA; 18 Department of Anatomy and Neurosciences, Amsterdam UMC (location VUmc), Amsterdam, Holland Netherlands; 19 Department of Psychiatry, Amsterdam UMC (location VUmc), Amsterdam, Holland Netherlands; 20 Department of Psychiatry and Behavioral Sciences, Emory University, Atlanta, GA USA; 21 Department of Gynecology and Obstetrics, Emory University, Atlanta, GA USA; 22 Genomics Program, University of South Florida College of Public Health, Tampa, FL USA

**Keywords:** EWAS, Longitudinal, DNA methylation, Meta-analysis, Trauma, PTSD, Epigenetics

## Abstract

**Background:**

Epigenetic mechanisms have been suggested to play a role in the development of post-traumatic stress disorder (PTSD). Here, blood-derived DNA methylation data (HumanMethylation450 BeadChip) collected prior to and following combat exposure in three cohorts of male military members were analyzed to assess whether DNA methylation profiles are associated with the development of PTSD. A total of 123 PTSD cases and 143 trauma-exposed controls were included in the analyses. The Psychiatric Genomics Consortium (PGC) PTSD EWAS QC pipeline was used on all cohorts, and results were combined using a sample size weighted meta-analysis in a two-stage design. In stage one, we jointly analyzed data of two new cohorts (*N* = 126 and 78) for gene discovery, and sought to replicate significant findings in a third, previously published cohort (*N* = 62) to assess the robustness of our results. In stage 2, we aimed at maximizing power for gene discovery by combining all three cohorts in a meta-analysis.

**Results:**

Stage 1 analyses identified four CpG sites in which, conditional on pre-deployment DNA methylation, post-deployment DNA methylation was significantly associated with PTSD status after epigenome-wide adjustment for multiple comparisons. The most significant (intergenic) CpG cg05656210 (*p* = 1.0 × 10^−08^) was located on 5q31 and significantly replicated in the third cohort. In addition, 19 differentially methylated regions (DMRs) were identified, but failed replication. Stage 2 analyses identified three epigenome-wide significant CpGs, the intergenic CpG cg05656210 and two additional CpGs located in *MAD1L1* (cg12169700) and *HEXDC* (cg20756026). Interestingly, cg12169700 had an underlying single nucleotide polymorphism (SNP) which was located within the same LD block as a recently identified PTSD-associated SNP in *MAD1L1*. Stage 2 analyses further identified 12 significant differential methylated regions (DMRs), 1 of which was located in *MAD1L1* and 4 were situated in the human leukocyte antigen (HLA) region.

**Conclusions:**

This study suggests that the development of combat-related PTSD is associated with distinct methylation patterns in several genomic positions and regions. Our most prominent findings suggest the involvement of the immune system through the HLA region and *HEXDC*, and *MAD1L1* which was previously associated with PTSD.

## Background

Post-traumatic stress disorder (PTSD) is a debilitating psychiatric disorder that can develop following direct or indirect exposure to a potentially life-threatening traumatic incident. Symptoms include persistent re-experiencing of the trauma, avoidance behavior, hyperarousal, and negative mood [1]. Although most individuals have the potential to withstand negative effects of trauma exposure on long-term mental health and to recover promptly, some are more vulnerable and at increased risk of developing PTSD. Understanding the molecular and neurobiological underpinnings of this differential susceptibility is currently receiving considerable attention, and epigenetic mediation of environmental influences has been proposed as a potential key mechanism [2–4].

Several epigenome-wide association studies (EWAS) have aimed to identify differentially methylated CpGs in PTSD [5–8]. However, most of these studies are based on association analyses where methylation was assessed at a single time point (cross-sectional), with limited ability to adjust for confounding variables. Only one PTSD study to date reported longitudinal changes in methylation profiles across a period of combat exposure in order to capture changes in DNA methylation over time in relation to phenotypic changes [7]. We made use of the Prospective Research In Stress-related Military Operations (PRISMO) study, which in the present study was used as a replication cohort.

Here, we followed a previously published two-stage design [9] where we first meta-analyzed two longitudinal, USA-based military cohorts in order to identify associations between changes in methylation levels from pre-deployment to post-deployment and the development of PTSD. We then sought replication of our significant findings in PRISMO. In the second stage, we combined all three cohorts in a meta-analysis. This two-stage approach allows us to investigate the robustness of our findings through replication in stage 1, while increasing power for gene discovery by combining all three studies in stage 2. For all three cohorts, DNA methylation data and phenotypic data were collected prior to and following a 4–7-month deployment to an active ware zone in Iraq or Afghanistan. All studies selected PTSD cases and controls at post-deployment and only included subjects without PTSD at pre-deployment. Of the significant CpGs in the second analysis stage, we assessed associations with nearby single nucleotide polymorphisms (SNPs) and gene expression data, and examined correlations between blood and brain methylation status. To the best of our knowledge, this is the largest study aimed at detecting methylation changes associated with the development of PTSD. This prospective and longitudinal analysis permits us to more accurately capture dynamic changes in DNA methylation in relation to PTSD development while minimizing confounding due to intra-individual variability.

## Results

### Cohorts

Three military cohorts were included in this study, i.e., the US Marine Resiliency Study (MRS), the US Army Study to Assess Risk and Resilience in Servicemembers (Army STARRS), and the Dutch PRISMO study. Demographic and clinical characteristics of subjects from all three cohorts (total *N* subjects = 266) can be found in Table 1. All subjects were male, and the majority were of European ancestry (*N* = 211, 79%). Within each cohort, cases and controls did not differ significantly in terms of age. Pre-deployment PTSD symptoms were significantly different between cases and controls from MRS only, with cases scoring slightly higher on the Clinician Administered PTSD Scale (CAPS) as compared to controls (*p* = .002; Table 1). In MRS and Army STARRS, cases were exposed to more traumatic events as compared to controls (*p* < .001 for both cohorts).

**Table 1:** Demographics and clinical characteristics of MRS, Army STARRS, and PRISMO

	Cases	Controls	*p* value	Overall
Number				
MRS	63	63	**–**	126
Army STARRS	31	47	**–**	78
PRISMO	29	33	**–**	62
Age, mean (SD)				
MRS	22.15 (2.3)	22.36 (3.7)	.71	22.26 (3)
Army STARRS	23.5 (4.0)	24.6 (4.8)	.26	24.2 (4.4)
PRISMO	27.1 (9.9)	27.1 (8.7)	1.0	27.1 (9.0)
PTSD pre-deployment, mean (SD)				
MRS, CAPS	10.8 (7.5)	6.8 (6.5)	.002	8.8 (7)
Army STARRS, PCL-6	7.4 (2.6)	6.8 (2.0)	.40	7.0 (2.2)
PRISMO, SRIP	28.2 (4.0)	26.4 (4.0)	.10	27.2 (3.9)
PTSD post-deployment, mean (SD)				
MRS, CAPS	58.17 (13.5)	13.36 (6.1)	< .001	35.76 (9.8)
Army STARRS, PDL-C	52.7 (7.8)	25.8 (8.6)	< .001	36.5 (8.1)
PRISMO, SRIP	46.1 (8.7)	27.4 (5.1)	< .001	36.1 (6.5)
Combat exposure, mean (SD)				
MRS, DDRI	1.08 (0.8)	0.66 (0.4)	< .001	0.87 (0.6)
Army STARRS, PCL	9.4 (1.3)	7.9 (2.0)	< .001	8.5 (1.7)
PRISMO, DEC	8.5 (3.0)	7.2 (2.3)	.07	7.8 (2.5)
Ancestry, *N* (%)				
MRS				
European	34 (53)	37 (59)	–	71 (56)
African	5 (8)	5 (8)	–	10 (8)
Other	24 (39)	21 (33)	–	45 (36)
Army STARRS				
European	31 (100)	47 (100)	–	78 (100)
PRISMO				
European	29 (100)	33 (100)	–	62 (100)

*CAPS* Clinician-Administered PTSD Scale, *PCL-6* PTSD Checklist—screener, *SRIP* Self-Report Inventory for PTSD, *PCL-C* PTSD Checklist—civilian version, *DDRI* Deployment Risk and Resilience Inventory, *DEC* deployment experiences checklist, *SD* standard deviation. Each study used different scales for PTSD and combat exposure scores; the corresponding scales are included in the row names

### Stage 1: meta-analysis of MRS and Army STARRS

Data from MRS and Army STARRS were combined in order to identify CpG sites in which, conditional on baseline DNA methylation, post-deployment methylation was associated with PTSD status. Four genome-wide significant CpG sites (i.e., differentially methylated positions, DMPs) were identified using a conservative Bonferroni threshold of *p* = 1.13 × 10^−07^ for the 450K EWAS array (Table 2). These sites were located near *SPRY4*, in *SDK1*, *CTRC*, and *CDH15*. The direction of DNA methylation profiles associated with PTSD development was different for each site (Additional file 1: Figures S1-4). Additionally, after the Bonferroni correction for ~ 26,000 predefined regions, 19 differentially methylated regions (DMRs) were identified in which, conditional on baseline DNA methylation, post-deployment methylation was significantly associated with PTSD status (Table 3).

**Table 2:** Differentially methylated positions (DMPs) in MRS, Army STARRS, and PRISMO

Probe	Chr: position	Gene	Region	MRS			Army STARRS			Stage 1: MRS and Army STARRS		Stage 1: replication in PRISMO			Stage 2: meta-analysis of 3 cohorts	
				β	SE	*p* value	β	SE	*p* value	*Z*	*p* value	β	SE	*p* value	*Z*	*p* value
cg05656210	5: 141660565	Intergenic	Intergenic	− 0.37	0.15	1.6E−02	− 0.58	0.09	6.1E−10	− 5.73	*1.0E−08*	− 0.47	0.20	2.0E−02*	− 6.14	*8.1E−10*
cg12169700	7: 1923695	*MAD1L1*	Body	− 1.24	0.27	4.2E−06	− 0.19	0.20	3.3E−01	− 4.22	2.4E−05	− 0.64	0.14	4.3E−06	− 5.91	*3.3E−09*
cg20756026	17: 80394529	*HEXDC*	Body	− 0.62	0.21	3.3E−03	− 0.28	0.09	2.6E−03	− 4.17	3.0E−05	− 0.37	0.09	2.6E−05	− 5.69	*1.3E−08*
cg16956686	7: 4304779	*SDK1*	Body	− 0.19	0.04	3.6E−07	− 0.13	0.05	7.1E−03	− 5.67	*1.5E−08*	− 0.04	0.09	6.3E−01	− 5.20	2.0E−07
cg18917957	1: 15764093	*CTRC*	TSS1500	− 0.34	0.08	2.3E−05	− 0.26	0.08	4.9E−04	− 5.48	*4.2E−08*	− 0.06	0.13	6.4E−01	− 5.03	5.0E−07
cg05901543	16: 89251975	*CDH15*	Body	− 0.14	0.03	2.4E−08	− 0.06	0.03	7.1E−02	− 5.50	*3.7E−08*	0.01	0.05	8.2E−01	− 4.71	2.5E−06

*SE* standard error. All positions and regions were in reference to GRCh37/hg19. Significance (*p* < 1.13 × 10^−7^) is indicated in italics. The asterisk indicates significance of replication after the Bonferroni correction for four probes (one-sided *z* test). The *p* values for MRS, Army STARRS, and the combined analyses are Bonferroni-corrected for ~ 450K CpG sites. In stage 1, MRS and Army STARRS were combined and PRISMO was used to replicate significant findings. In stage 2, all three studies were combined. The table is organized based on significances of the DMPs in the stage 2 meta-analysis

**Table 3:** Differentially methylated regions (DMRs) in MRS, Army STARRS, and PRISMO

Chr: start-stop	No. of probes	Gene	Region	MRS		Army STARRS		Stage 1: MRS and Army STARRS		Stage 1: replication in PRISMO		Stage 2: meta-analysis of 3 cohorts	
				NES	*p* value	NES	*p* value	*Z*	*p* value	NES	*p* value	*Z*	*p* value
6: 33043976-33054001	56	*HLA-DPB1*	Body	− 2.08	3.45E−05	− 1.98	8.06E−05	− 5.69	*1.25E−08*	− 1.05	3.51E−01	− 5.43	*5.46E−08*
6: 33048416-33048814	17	*HLA-DBP1*	Island	− 2.04	6.96E−05	− 2.25	1.62E−04	− 5.46	*4.80E−08*	− 1.16	2.15E−01	− 5.38	*7.49E−08*
21: 35831697-35832365	10	*KCNE1*	Island	− 1.93	1.32E−04	− 2.16	1.72E−04	− 5.33	*9.99E−08*	− 1.27	1.58E−01	− 5.34	*8.93E−08*
21: 35827824-35884508	23	*KCNE1*	Promoter	− 1.93	9.45E−05	− 2.00	3.42E−04	− 5.28	*1.27E−07*	− 1.22	1.91E−01	− 5.26	*1.46E−07*
6: 32547019-32557404	34	*HLA-DRB1*	Body	− 1.23	2.00E−01	− 2.48	1.67E−05	− 3.67	2.43E−04	− 2.58	2.62E−05	− 5.24	*1.58E−07*
7: 1885033-1885402	3	*MAD1L1*	Island	− 2.22	1.81E−05	− 2.12	1.72E−04	− 5.69	*1.25E−08*	− 0.81	7.38E−01	− 5.15	*2.65E−07*
7: 27169572-27170638	10	*HOXA4*	Island	− 2.21	1.73E−05	− 1.98	3.26E−04	− 5.60	*2.15E−08*	− 0.84	7.46E−01	− 5.06	*4.20E−07*
8: 125461772-125464547	9	*TRMT12*	Promoter	− 2.20	1.85E−05	− 1.14	2.98E−01	− 4.01	6.11E−05	− 1.93	1.55E−03	− 5.03	*4.69E−07*
6: 32551851-32552331	13	*HLA-DRB1*	Island	− 1.25	1.79E−01	− 2.49	1.68E−04	− 3.38	7.17E−04	− 2.52	2.59E−05	− 4.99	*5.92E−07*
7: 27169740-27171528	24	*HOXA4*	Promoter	− 2.31	1.78E−05	− 1.97	3.41E−04	− 5.59	*2.31E−08*	− 0.68	9.18E−01	− 4.94	*7.71E−07*
6: 25882327-25882560	4	*SLC17A3*	Island	− 1.96	9.39E−05	− 1.50	4.91E−02	− 4.29	1.81E−05	− 1.63	3.04E−02	− 4.80	*1.60E−06*
1: 156814881-156815792	5	*NTRK1*	Island	− 1.51	5.29E−02	− 1.78	3.74E−03	− 3.31	9.20E−04	− 2.12	1.16E−04	− 4.76	*1.90E−06*
16: 1561036-1652552	121	*IFT140*	Body	− 1.67	7.56E−04	− 1.82	5.99E−05	− 5.12	*2.92E−07*	− 0.83	8.80E−01	− 4.57	4.96E−06
17: 8700574-8703341	12	*MFSD6L*	Promoter	− 2.33	1.84E−05	− 1.82	3.56E−03	− 5.17	*2.36E−07*	1.03	4.10E−01	− 4.52	6.33E−06
16: 1583809-1584641	8	*IFI140*	Island	− 2.46	1.81E−05	− 1.79	2.75E−03	− 5.22	*1.78E−07*	0.69	8.80E−01	− 4.50	6.79E−06
5: 191792-192544	5	*LRRC14B*	Island	− 2.19	1.81E−05	− 1.57	2.34E−02	− 4.77	*1.83E−06*	− 0.93	5.50E−01	− 4.47	7.96E−06
6: 168433191-1.68E+08	18	*KIF25*	Body	− 2.13	5.45E−05	− 1.79	4.11E−03	− 4.94	*7.59E−07*	− 0.62	9.50E−01	− 4.36	1.30E−05
10: 530713-531099	5	*DIP2C*	Island	− 2.22	3.54E−05	− 1.98	5.05E−04	− 5.40	*6.63E−08*	1.148	2.60E−01	− 4.19	2.78E−05
1: 2986362-3349982	608	*PRDM16*	Body	− 1.73	1.34E−05	− 1.43	2.06E−04	− 5.71	*1.09E−08*	1.168	8.00E−02	− 4.15	3.25E−05
17: 8702342-8702824	7	*MFSD6L*	Island	− 2.19	1.84E−05	− 1.82	2.42E−03	− 5.24	*1.59E−07*	0.684	8.70E−01	− 4.13	3.57E−05
12: 9217328-9217715	6	*LINC00612*	Island	− 2.27	1.84E−05	− 2.36	1.75E−04	− 5.69	*1.30E−08*	1.465	6.00E−02	− 4.09	4.31E−05
12: 9217079-9217769	9	*LOC144571*	Promoter	− 2.27	1.85E−05	− 2.16	1.81E−04	− 5.68	*1.34E−08*	1.530	4.00E−02	− 4.00	6.46E−05
11: 70672834-70673055	6	*SHANK2*	Island	− 2.56	1.83E−05	− 2.32	1.73E−04	− 5.69	*1.28E−08*	1.752	6.00E−03	− 3.67	2.45E−04
17: 76037074-76037323	3	*TNRC63*	Island	− 2.03	9.29E−05	− 2.05	1.78E−04	− 5.39	*7.07E−08*	1.693	1.10E−02	− 3.49	4.82E−04

*Chr* chromosome, *NES* normalized effect score. All positions and regions were in reference to GRCh37/hg19. Significance is indicated in italics. The *p* values for MRS, Army STARRS, and the combined analyses are Bonferroni-corrected for ~ 26K DMRs. In stage 1, MRS and Army STARRS were combined and PRISMO was used to replicate significant findings. In stage 2, all three studies were combined. The table is organized based on significances of the DMRs in the stage 2 meta-analysis

### Replication in PRISMO

After the Bonferroni correction for the four significant DMPs and when using a one-sided test, the association of one CpG site, the intergenic site cg05656210, was replicated in PRISMO (*p* = 2.0 × 10^−02^; Table 2). Both the discovery meta-analysis and replication analysis show decreased DNA methylation in association with PTSD status. None of the 19 significant DMRs were replicated in PRISMO (Table 3).

### Stage 2: meta-analysis of MRS, Army STARRS, and PRISMO

**Fig. 1:**
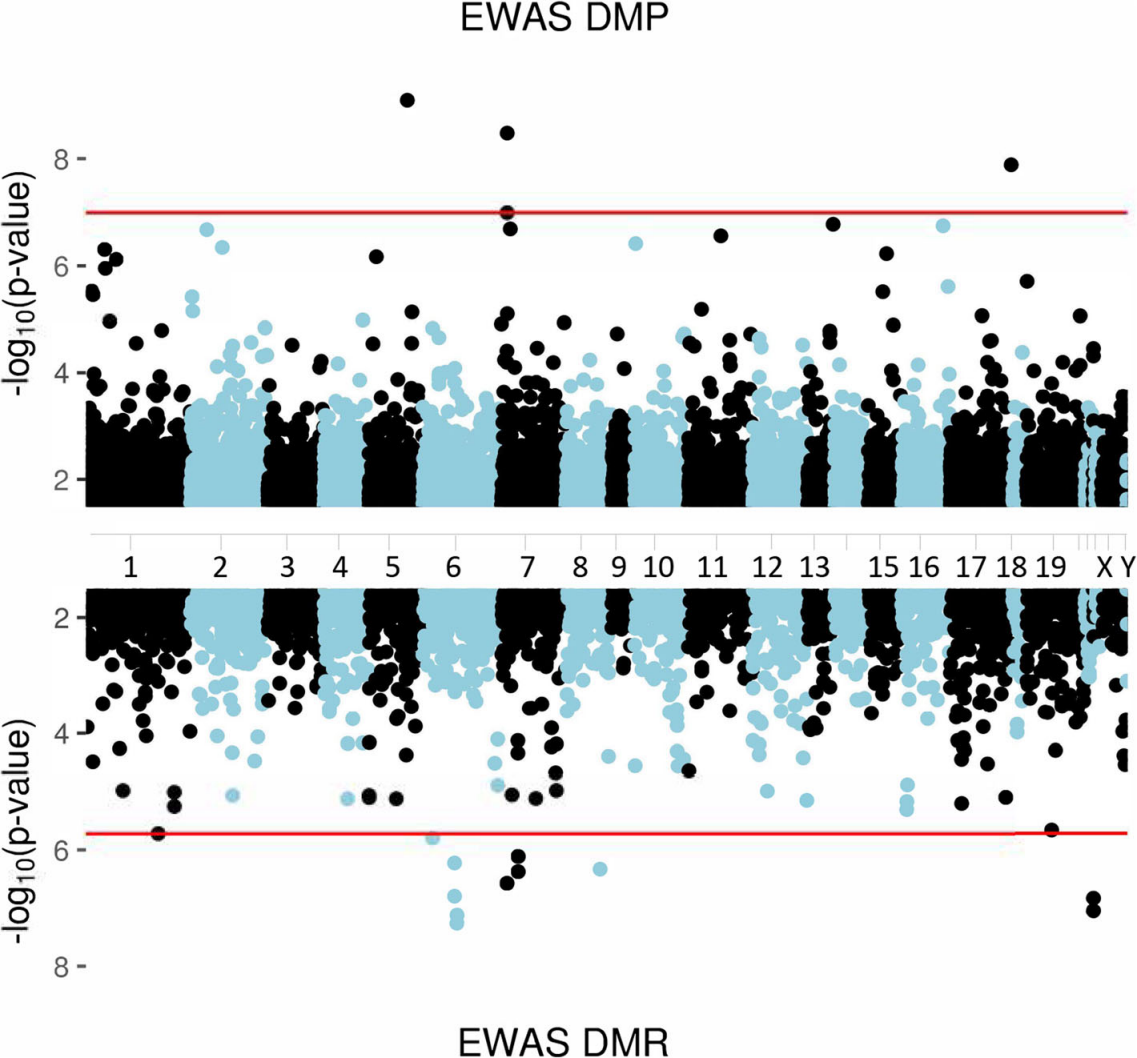
Manhattan plot showing the results of the stage 2 meta-analysis across 3 epigenome-wide association studies (MRS, Army STARRS, PRISMO). The upper part shows the 3 significant differentially methylated positions (DMPs) while the lower part shows the 12 significant differentially methylated regions (DMRs). Red lines indicate significance thresholds after the Bonferroni corrections for ~ 485,000 (top) and 26,000 (bottom) comparisons, respectively

When combining MRS, Army STARRS, and PRISMO, the DNA methylation profile of three CpG sites was significantly associated with post-deployment PTSD status (Table 2, Fig. 1). The intergenic CpG that replicated in PRISMO remained the most significant (*Z* = − 6.14, *p* = 8.1 × 10^−10^). The other sites were located in the gene body regions of *MAD1L1* and *HEXDC* (Additional file 1: Figures S1, S5, S6). Sensitivity analyses for the potentially confounding effects of changes in smoking and alcohol use did not substantially affect these results (Additional file 1: Table S1). Furthermore, 12 DMRs were identified (Additional file 1: Figures S7-18, Fig. 1), 7 of which were also significant in stage 1 and 4 were located in the human leukocyte antigen (HLA) region (Table 3).

#### Genetic effects and gene expression

Using MRS data, genetic effects on DNA methylation levels of the significant DMPs were assessed by testing for associations with SNPs within 500 kb of the DMPs. All DMPs had significantly associated SNPs which explained approximately 80% of the variation in methylation (*p* < 2 × 10^−16^) and were located within 1 bp of their respective CpG sites (Additional file 1: Table S2). However, adjusting for genotypes in the main model to assess the impact of SNPs on the association between DNA methylation and PTSD did not substantially affect the observed findings (Additional file 1: Table S3). We further assessed the association between baseline or post-deployment methylation signatures of these DMPs and corresponding blood-derived gene expression data which was available for MRS [10]. At baseline, methylation levels of the CpGs located in *HEXDC* and *MAD1L1* were significantly correlated with gene expression data (Table 4), with an inverse correlation between DNA methylation and expression in *HEXDC* and a positive correlation between methylation and expression of *MAD1L1*. Post-deployment DNA methylation values correlated to gene expression of *HEXDC* only. Methylation of the intergenic site cg05656210 was not significantly associated with expression of the closest gene at any time point. We additionally assessed the association between changes in expression and changes in methylation for these DMPs and found no significant associations (Table 4).

**Table 4:** Correlations between methylation levels of DMPs and gene expression data from MRS

CpG	Gene	Baseline corr (*r*)	*p* value	Post-deployment corr (*r*)	*p* value	Change corr (*r*)	*p* value
cg20756026	*HEXDC*	− 0.28	*2.00E−03*	− 0.24	*8E−03*	− 0.01	0.88
cg12169700	*MAD1L1*	0.23	*1.00E−02*	0.06	0.49	− 0.07	0.46
cg05656210	SPRY4*	0.08	3.80E−01	0.11	0.21	0.10	0.27

Significance is indicated in italics. *Corr* correlation

*Closest gene

#### Blood-brain correlations of PTSD-associated CpGs

Blood-brain correlations of methylation levels of the significant stage 2 DMPs were examined using a publicly available database [11]. For all three DMPs, blood DNA methylation levels correlated strongly with those in the prefrontal cortex, entorhinal cortex, superior temporal gyrus, and cerebellum (*r ≥* 0.93 for all sites; *p* values ranging between 1.48 × 10^−32^ and 5.32 × 10^−72^; Additional file 1: Table S4, Additional file 1: Figure S19 for cg05656210).

## Discussion

Exposure to trauma is a prerequisite for the development of PTSD, yet not all individuals develop PTSD following trauma [12]. The underlying biological mechanisms of this differential susceptibility have not yet been fully identified, and even the largest genome-wide association studies (GWAS) to date explain only a small proportion of the disease liability [13, 14]. Epigenetic changes have been studied as one potential mechanism, but most association studies have used cross-sectional designs which render it impossible to establish causality. Here, we use a more powerful longitudinal design to investigate associations of DNA methylation with post-deployment PTSD status across very similar military cohorts deployed to combat in Iraq and Afghanistan. We started with combining the USA-based MRS and Army STARRS cohorts and sought replication using the previously published Dutch PRISMO study [7]. To maximize power for new discoveries, we also performed a meta-analysis across all three cohorts. The first analysis stage revealed 4 genome-wide significant DMPs and 19 DMRs which were linked to post-deployment PTSD status. One of these DMPs replicated in PRISMO. In the second stage, a meta-analysis of all 3 studies revealed that the replicating DMP and 7 DMRs remained significant, and 2 additional DMPs and 12 DMRs were identified.

The replicating DMP cg05656210 remained the top-ranked significant marker in the second analysis stage. This CpG site is an intergenic site annotated near *SPRY4*. SPRY4 is a member of the Sprouty proteins which are mainly involved in inhibiting receptor tyrosine kinase (RTK) signaling [15]. Upon activation by growth factor ligands, RTK signaling has a wide variety of downstream effects ranging from the regulation of cell proliferation and differentiation to the modulation of cellular metabolism [16]. One particular receptor involved in RTK signaling, i.e., receptor tyrosine kinase B (TrkB), and its main ligand, brain-derived neurotrophic factor (BDNF), have repeatedly been shown to be affected in stress-related disorders such as depression [17]. Consistently, two independent studies reported decreased mRNA levels of BDNF and TrkB in the prefrontal cortex and hippocampus of individuals who committed suicide as compared to healthy control subjects [18, 19]. Moreover, SPRY4-IT1, a long non-coding RNA derived from the second intron of *SPRY4* [20], has been shown to interact with *SKA2* [21], a gene that was suggested to be a promising biomarker for suicidal behavior [22, 23], stress susceptibility, and stress-related disorders such as PTSD [23–25]. Finally, *SPRY4* was previously found differentially methylated in the blood of patients diagnosed with schizophrenia [26]. Together, these results suggest that alterations within *SPRY4* could contribute to psychiatric disorders such as depression, PTSD, and schizophrenia and potentially play a role in suicidal behavior. The question as to whether and how the identified DMP influences the expression of *SPRY4* and is involved in these phenotypes still remains to be answered.

The second top significant probe, cg12169700, and one DMR are located within *MAD1L1*. MAD1L1 is part of the mitotic spindle-assembly checkpoint (SAC) which monitors the proper attachment of chromosomes to the microtubule spindle apparatus, delays the start of anaphase until all chromosomes are properly attached, and in doing so, ensures correct chromosome separation [27, 28]. Malfunctions of the MAD1L1 protein could therefore contribute to aneuploidy and chromosomal instability. Specific SNPs within this gene have previously been associated with bipolar disorder [29, 30], schizophrenia [30–32], and depression [33]. Interestingly, *MAD1L1* was recently identified in a PTSD GWAS of the Million Veteran Program (MVP) [34]. The SNP that underlies cg12169700, rs11761270, is located in the same large linkage disequilibrium (LD) block as the MVP finding. In the MVP, carriers of the minor allele of rs11761270 showed decreased levels of methylation and were at increased risk of having PTSD. This corresponds to our own findings in which PTSD cases show a reduction in methylation from pre- to post-deployment. Moreover, using expression data from MRS, we found that methylation at this site was positively associated with gene expression of *MAD1L1*. This also aligns with previous findings that showed that blood levels of *MAD1L1* were decreased in highly stress-susceptible individuals [35]. Together, these findings suggest that specific methylation profiles within *MAD1L1* may be regarded as a risk factor for PTSD in addition to several other psychiatric disorders [36]. However, the underlying mechanisms through which such disturbances could contribute to psychiatric disorders warrant further research.

The third CpG site is located in *HEXDC* which to date has no known implications in any psychiatric disease. Although the biological functions of its product, hexosaminidase D, remain largely unknown, it is believed to be a glycosidase and previous studies found associations with rheumatoid arthritis [37, 38]. Interestingly, recent studies found significant pleiotropy between rheumatoid arthritis and PTSD [39], and rheumatoid arthritis and schizophrenia [40]. Other studies found associations between PTSD symptoms and rheumatoid arthritis in a twin population [41] and in an epidemiological study of military veterans [42]. This link between mental disorders and immune-related processes is discussed more in detail below. The DMP of *HEXDC* was located directly adjacent to rs4789774, a known expression quantitative trait locus (eQTL) that regulates the expression of *HEXDC* in the human brain cortex and of *NARF* and *NARF-IT1* in a number of tissue types including blood (http://genome.ucsc.edu/). Moreover, a modest negative correlation was found between methylation of this site and gene expression of *HEXDC*, both at baseline and at post-deployment.

Twelve significant DMRs were found in the second phase of the analysis. Our strongest finding was in the HLA region, a gene-dense region which contains over 200 genes that encode human leukocyte antigen complex proteins in charge of presenting peptide antigens to trigger immune reactions, among other (non-)immune functions [43]. Their non-immunological roles include processes such as neurodevelopment, synaptic plasticity, learning, memory, and stress reactivity [44, 45]. It is therefore not entirely surprising that several epigenetic modifications and genetic variants within this region have repeatedly been found implicated in neuropsychiatric disorders such as schizophrenia and bipolar disorder (recently reviewed in [46]). Along with our *HEXDC* finding, these observations further enhance the existing notion that immune factors play an important role and should continue to be studied in relation to mental disorders such as PTSD [47]. Although it is now clear that immune imbalances are present in PTSD, questions related to causality and further implications for prevention strategies and treatment options largely remain to be answered.

Follow-up analyses were done using the significant DMPs from the stage 2 meta-analysis only. The discovery that methylation levels at the top three PTSD-associated CpGs were highly associated with the genotype of the nearby SNPs led us to question whether the associations between methylation and PTSD status were mainly driven by genotype. However, direct adjustment for genotype in a sensitivity analysis did not attenuate the associations between DNA methylation and PTSD status and our current sample size limits our ability to conduct analyses specific to genotype strata to further investigate interaction effects between SNPs and methylation.

Since our methylation data were based on DNA from peripheral blood, we further examined correlations between blood and several brain regions, i.e., the prefrontal cortex, entorhinal cortex, superior temporal gyrus, and cerebellum. The results indicate that blood-brain correlations of all top DMPs were strong for all four brain regions suggesting that these findings could potentially also be relevant for tissues other than blood. Assessing these correlations is relevant when dealing with disorders such as PTSD which are characterized by functional and structural alterations within the brain but for which the accessibility to human brain tissue is limited. However, these and similar findings will need to be confirmed using postmortem brain tissue and their precise role in PTSD development will need to be investigated further.

The main limitation of the present study is its small sample size which likely captures only a fraction of all implicated CpGs and renders additional analyses such as pathway and network analyses underpowered. It further needs to be emphasized that this study used data generated with Illumina’s 450K arrays which only assess a subset of all CpG sites. Next, although examining blood-derived DNA methylation is informative when seeking relatively easily accessible biomarkers, follow-up studies are needed in order to assess these methylation patterns within the tissue of interest, i.e., the brain. Another important limitation of the study is the fact that subjects that developed PTSD were exposed to significantly more traumatic events than control subjects in MRS and Army STARRS. One way to address these differences would be to include trauma exposure as a covariate. However, given the high correlation between trauma exposure and PTSD, this would likely diminish the association of methylation changes and PTSD. Therefore, we acknowledge that our analyses may include associations with trauma exposure in addition to PTSD. Next, the limited replication of findings from the US cohorts in the Dutch PRISMO study, and vice versa [7], may point to type I errors, or be partially due to small sample sizes and/or heterogeneity in study designs, study environments, and potential confounders such as immune status or medication use. For example, findings reported from PRISMO [7] point towards the involvement of DMPs and DMRs located within *ZFP57*, *RNF39*, and *HIST1H2APS2*, which were not implicated in our analyses. However, these genes are located within the HLA region, a region which is indeed implicated in our analyses. Furthermore, whereas the PRISMO study is entirely based on subjects of European ancestry, the US MRS and Army STARRS studies include more ancestral diversity. Genes in the HLA region tend to be highly polymorphic, particularly with respect to ancestral background [48]. Thus, it is possible that this heterogeneity may contribute to the lack of replication. Further analyses should be ancestry-specific, once sample sizes are adequate. Furthermore, at this stage, it is unclear whether the identified differential methylation patterns in PTSD cases have any functional consequences. Although they may influence gene expression, the current dataset has limited power to establish causality. In order to make claims regarding causation, performing functional studies in vitro and animal studies will be needed in order to unravel precise biological mechanisms. Finally, to maximize power for discovery, the present cohorts were chosen to be highly similar in regard to demographics, type of trauma, and time since trauma exposure. Thus, the degree to which these findings on active duty, predominantly European-ancestry military men, may generalize to females, civilians, or other ancestries is unclear.

## Conclusions

In summary, this is the largest study on methylation changes associated with the development of combat-related PTSD to date. Our observations point towards the implication of biologically interesting genes such as HLA region which is involved in immune-related processes, *HEXDC* which also has been suggested to play a role in immunity, and *MAD1L1*, a PTSD-related gene recently identified in the large MVP. These findings strengthen the notion that specific DNA methylation profiles are involved in the development of combat-related PTSD. Larger longitudinal studies and integrative efforts are now needed to build upon these preliminary findings in order to understand their functional consequences and integrate them more broadly into our current understanding of the (epi)genomic basis of PTSD.

## Methods

### Discovery datasets

#### Marine Resiliency Study

The Marine Resiliency Study (MRS) [49] is a prospective PTSD study of Marines and Navy personnel deployed to Iraq or Afghanistan. PTSD symptoms were assessed approximately 1 month before deployment, and 3 and/or 6 months post-deployment using the CAPS and the PTSD Checklist (PCL) for DSM-IV. Biological samples including whole blood were collected at all time points. Information on smoking and alcohol use was collected on a self-report basis. Combat exposure was assessed approximately 1 week post-deployment using the Deployment Risk and Resilience Inventory (DRRI). A subset of 63 PTSD cases and 63 controls was selected for the methylation assays and inclusion in the present study. All subjects were free of a PTSD diagnosis at pre-deployment and had CAPS scores ≤ 25. After return from a ~ 7-month deployment period, blood samples from PTSD cases (following the DSM-IV full or partially stringent diagnosis [50, 51]) were selected either at the 3- or the 6-month follow-up visit, based on when these subjects had their highest recorded CAPS scores. Subsequently, controls were frequency matched to the selected cases for age, ancestry, and time of post-deployment visit. The study was approved by the institutional review boards of the University of California San Diego, VA San Diego Research Service, and Naval Health Research Center. All subjects provided informed consent.

#### Army STARRS

The Army Study to Assess Risk and Resilience in Servicemembers (Army STARRS) is a prospective study among US Army personnel gathering information on risk and resilience factors for suicidality and psychopathology [52]. All subjects completed a computerized version of the Composite International Diagnostic Interview screening scales (CIDI-SC) and the PCL6 screener for DSM-IV approximately 6 weeks before deployment to Afghanistan, and the PCL-C at 1, 2, and 6 months post-deployment. PTSD diagnosis was assigned using multiple imputation methods that relied on PCL and CIDI-SC data [53], and information on trauma exposure was gathered from self-administered questions on childhood-, adult-, and military-related events. Information on smoking and alcohol use was collected on a self-report basis. Biological samples including whole blood were collected approximately 6 weeks before deployment and 1 month post-deployment. A subset of 31 cases and 47 controls were selected for the methylation assays and inclusion in this analysis. All subjects were free of a PTSD diagnosis at pre-deployment. PTSD cases were selected based on their PTSD diagnosis at 6 months post-deployment. Controls were PTSD-free subjects matched on age, deployment stress, and childhood adversity. The study procedures were approved by the Institutional Review Boards of all collaborating organizations. All subjects provided informed consent.

#### Replication dataset: PRISMO

Replication data was obtained from the Prospective Research In Stress-related Military Operations (PRISMO) study, a prospective study of Dutch military soldiers deployed to Afghanistan [54, 55]. The severity of current PTSD symptoms was assessed using the Self-Report Inventory for PTSD (SRIP), and blood samples were collected approximately 1 month before and 1 and 6 months after deployment. Traumatic stress exposure during deployment to Afghanistan was assessed with a deployment experiences checklist. Information on smoking and alcohol use was collected on a self-report basis. A subset of 29 cases and 33 controls was selected for the methylation assays and inclusion in this analysis (see [7] for selection criteria). The study was approved by the ethical committee of the University Medical Center Utrecht, and was conducted in accordance with the Declaration of Helsinki. All subjects provided informed consent.

### Quality control

In all cohorts, longitudinal whole blood DNA methylation levels were measured using the Illumina HumanMethylation450K BeadChip. The Psychiatric Genomics Consortium (PGC)-EWAS quality control pipeline was used on all three cohorts [5]. Briefly, samples were excluded when having a probe detection call rate < 90% and an average intensity value < 50% of the overall sample mean or < 2,000 arbitrary units (AU). Individual probes with detection *p* values > 0.001 or those based on less than three beads were set to missing. Remaining probes were excluded when cross-reactivity occurred between autosomal and sex chromosomes. CpG sites with missing data for > 10% of samples within cohorts were excluded (Additional file 1: Table S5). After filtering, the β-values reflecting methylation levels of individual cytosine residues were normalized to correct for differences between type I and type II probes using Beta Mixture Quantile Normalization (BMIQ) [56]. ComBat [57] was used to correct for remaining issues such as batch and plate effects. To account for differences in cell type composition between samples, proportions of CD8, CD4, NK, B cells, monocytes, and granulocytes were estimated for each individual using their unique DNA methylation profiles. This was estimated using the estimatecellcounts function in minfi [58].

### Statistical analysis

The normalized β-values were logit transformed to *M* values which were used for linear regression analysis. Post-deployment DNA methylation was modeled as a function of post-deployment PTSD status while adjusting for pre-deployment DNA methylation; age; changes in CD4T, CD8T, NK, B cell, and monocyte cell proportions; and principal components (PCs) for ancestry. For MRS and Army STARRS, the PCs were derived from available GWAS and PCs 1–3 were included. For PRISMO, the method described by Barfield and colleagues [59] was used to derive PCs from the EWAS data and PCs 2–4 (see [5]) were included. HC3 standard errors were calculated using the sandwich R library [60]. Analyses were performed on each cohort independently, and the obtained *p* values were combined using a sample size weighted meta-analysis. Significance was declared at *p* < 1.13 × 10^−7^ after a stringent Bonferroni correction for 439,897 probes. Possible confounding effects of changes in smoking and alcohol use were assessed as a sensitivity analysis.

DMR analysis was performed on a set of 26,000 pre-defined gene regions within gene bodies, promoter regions, and CpG islands using the mCSEA version 1.2 package for R [61]. Regions were included when annotated to having at least five CpGs. For each study, EWAS *p* values, methylation level values, and a phenotype and covariate data matrix were supplied as program inputs. *p* values were derived using 100,000 permutations. A sample size weighted meta-analysis of DMRs was performed based on *z*-score transformations of permutation *p* values. Significances of DMRs (*p* < 1.92 × 10^−6^) were derived based on a Bonferroni correction for the 26,000 tests performed. All positions and regions were in reference to GRCh37/hg19.

We considered replication as significant when the effect directions matched between the discovery and replication samples and the *p* values held up to the Bonferroni correction for the number of replications attempted (i.e., 4 for the DMPs, 19 for the DMRs) using a one-sided test.

### Detecting genetic effects and links with gene expression

Associations between baseline levels of methylation of each significant CpG from the second analysis stage and nearby SNPs (within 500 kilobases; kb) were assessed in the MRS dataset using PLINK [62] to detect the potential influence of genetic effects on DNA methylation. For a given CpG site, the SNP with the lowest *p* value was carried forward as an additional covariate in the regression models as a sensitivity analysis.

For CpGs annotated to genes, we estimated the correlations between baseline or post-deployment CpG methylation levels and corresponding blood gene expression in MRS data. We additionally estimated the correlations between changes in expression and changes in methylation for these CpGs. Details of mRNA expression measurement in MRS can be found elsewhere [63]. Briefly, from 124 MRS Marine participants, blood was drawn at ~ 1 month pre-deployment and mRNA levels were analyzed through sequencing on a Illumina Hi-Seq 2000.

We used the UCSC genome browser tool (http://genome.ucsc.edu/) to identify if SNPs associated with our CpGs influenced expression in other tissue types based on combined expression eQTL data from 44 tissues from GTEx v6 [64].

### Blood-brain correlations

The Blood Brain DNA Methylation Comparison Tool (http://epigenetics.iop.kcl.ac.uk/bloodbrain/) was used to assess correlations between the methylation status of the top hits of the combined meta-analysis in blood and brain [11]. Specifically, this tool yields Pearson’s correlation coefficients (*r*) and associated *p* values for the association of the methylation status of individual CpG sites in blood and the prefrontal cortex, entorhinal cortex, superior temporal gyrus, and cerebellum.

## Supplementary information

**Additional file 1: Table S1.** Differentially methylated positions (DMPs) in MRS, Army STARRS and PRISMO with and without corrections for smoking status and alcohol use. **Table S2.** SNPs within 500 bps upstream or downstream of the significant DMPs. **Table S3.** Differentially methylated positions (DMPs) in MRS with and without correction for main associated SNPs. **Table S4.** Correlations between blood and brain methylation levels for the top CpG sites based on external data. **Table S5.** Number of probes and samples removed at each stage of the quality control pipeline. **Figure S1.** Methylation values (B values) at cg05656210 for each cohort separately. **Figure S2.** Methylation values (B values) at cg05901543 for each cohort separately. **Figure S3.** Methylation values (B values) at cg16956686 for each cohort separately. **Figure S4.** Methylation values (B values) at cg18917957 for each cohort separately. **Figure S5.** Methylation values (B values) at cg12169700 for each cohort separately. **Figure S6.** Methylation values (B values) at cg20756026 for each cohort separately. **Figure S7.** Differentially methylated region at HLA-DBP1: 33048416-33048814. **Figure S8.** Differentially methylated region at HLA-DPB1: 33043976-33054001. **Figure S9.** Differentially methylated region at HLA-DRB1: 32547019-32557404. **Figure S10.** Differentially methylated region at HLA-DRB1: 32551851-32552331. **Figure S11.** Differentially methylated region at HOXA4: 27169572-27170638. **Figure S12.** Differentially methylated region at HOXA4: 27169740-27171528. **Figure S13.** Differentially methylated region at KCNE1: 35827824-35884508. **Figure S14.** Differentially methylated region at KCNE1: 35831697-35832365. **Figure S15.** Differentially methylated region at MAD1L1: 1885033-1885402. **Figure S16.** Differentially methylated region at NTRK1: 156814881-156815792. **Figure S17.** Differentially methylated region at SLC17A3: 25882327-25882560. **Figure S18.** Differentially methylated region at TRMT12: 125461772-125464547. **Figure S19.** Example of blood-brain correlations of methylation levels in cg05656210. PFC: prefrontal cortex, EC: entorhinal cortex, STG: superior temporal gyrus, CER: cerebellum.

## Data Availability

Since the present study makes use of military datasets which are typically not shared publicly due to confidentiality reasons, the datasets used and/or analyzed during the current study are available from the corresponding author upon reasonable request only.

## Abbreviations

Army STARRS: Army Study to Assess Risk and Resilience in Servicemembers
BMIQ: Beta Mixture Quantile Normalization
CAPS: Clinician Administered PTSD Scale
DMP: Differentially methylated position
DMR: Differentially methylated region
DRRI: Deployment Risk and Resilience Inventory
eQTL: Expression quantitative trait locus
EWAS: Epigenome-wide association study
GWAS: Eenome-wide association studies
HLA: Human leukocyte antigen
LD: Linkage disequilibrium
MRS: Marine Resiliency Study
PCL: PTSD Checklist
PGC: Psychiatric Genomics Consortium
PRISMO: Prospective Research In Stress-related Military Operations
PTSD: Post-traumatic stress disorder
SNP: Single nucleotide polymorphism
SRIP: Self-Report Inventory for PTSD
