# Supplementary material for: Longitudinal epigenome-wide association studies of three male military cohorts reveal multiple CpG sites associated with post-traumatic stress disorder
Source: Clin Epigenetics. 2020 Jan 13;12:11. doi: 10.1186/s13148-019-0798-7 (PMC6958602; doi:10.1186/s13148-019-0798-7)
Supplement: Supplementary file 1 — Additional file 1: Table S1. Differentially methylated positions (DMPs) in MRS, Army STARRS and PRISMO with and without corrections for smoking status and alcohol use. Table S2. SNPs within 500 bps upstream or downstream of the significant DMPs. Table S3. Differentially methylated positions (DMPs) in MRS with and without correction for main associated SNPs. Table S4. Correlations between blood and brain methylation levels for the top CpG sites based on external data. Table S5. Number of probes and samples removed at each stage of the quality control pipeline. Figure S1. Methylation values (B values) at cg05656210 for each cohort separately. Figure S2. Methylation values (B values) at cg05901543 for each cohort separately. Figure S3. Methylation values (B values) at cg16956686 for each cohort separately. Figure S4. Methylation values (B values) at cg18917957 for each cohort separately. Figure S5. Methylation values (B values) at cg12169700 for each cohort separately. Figure S6. Methylation values (B values) at cg20756026 for each cohort separately. Figure S7. Differentially methylated region at HLA-DBP1: 33048416-33048814. Figure S8. Differentially methylated region at HLA-DPB1: 33043976-33054001. Figure S9. Differentially methylated region at HLA-DRB1: 32547019-32557404. Figure S10. Differentially methylated region at HLA-DRB1: 32551851-32552331. Figure S11. Differentially methylated region at HOXA4: 27169572-27170638. Figure S12. Differentially methylated region at HOXA4: 27169740-27171528. Figure S13. Differentially methylated region at KCNE1: 35827824-35884508. Figure S14. Differentially methylated region at KCNE1: 35831697-35832365. Figure S15. Differentially methylated region at MAD1L1: 1885033-1885402. Figure S16. Differentially methylated region at NTRK1: 156814881-156815792. Figure S17. Differentially methylated region at SLC17A3: 25882327-25882560. Figure S18. Differentially methylated region at TRMT12: 125461772-125464547. Figure S19. Example of blood-brai [file 13148_2019_798_MOESM1_ESM.docx]

**Supplemental tables and figures Snijders, Maihofer et al. Longitudinal epigenome-wide association studies of 3 male military cohorts reveal multiple CpG sites associated with post-traumatic stress disorder**

| **Supplemental Table S1**. Differentially methylated positions (DMPs) in MRS, Army STARRS and PRISMO with and without corrections for smoking status and alcohol use | | | | | | | | | | |
| --- | --- | --- | --- | --- | --- | --- | --- | --- | --- | --- |
|  |  | Stage 1: MRS and Army STARRS | | Stage 1: Replication in PRISMO | | | Stage 2: Meta-analysis of 3 cohorts | | Meta-analysis of 3 cohorts, corrected for smoking and alcohol use | |
| Probe | Gene | Z | *P-*value | β | SE | *P-*value | Z | *P*-value | Z | *P*-value |
| cg05656210 | *SPRY4* | -5.73 | **1.0E-08** | -0.47 | 0.20 | 2.0E-02* | -6.14 | **8.1E-10** | -6.50 | **1.9E-10** |
| cg12169700 | *MAD1L1* | -4.22 | 2.4E-05 | -0.64 | 0.14 | 4.3E-06 | -5.91 | **3.3E-09** | -5.00 | **6.1E-09** |
| cg20756026 | *HEXDC* | -4.17 | 3.0E-05 | -0.37 | 0.09 | 2.6E-05 | -5.69 | **1.3E-08** | -5.93 | **4.5E-10** |
| cg16956686 | *SDK1* | -5.67 | **1.5E-08** | -0.04 | 0.09 | 6.3E-01 | -5.20 | 2.0E-07 | -5.13 | 4.4E-08 |
| cg18917957 | *CTRC* | -5.48 | **4.2E-08** | -0.06 | 0.13 | 6.4E-01 | -5.03 | 5.0E-07 | -5.33 | 1.9E-07 |
| cg05901543 | *CDH15* | -5.50 | **3.7E-08** | 0.01 | 0.05 | 8.2E-01 | -4.71 | 2.5E-06 | -4.40 | 1.3E-06 |
| SE: standard error. All positions and regions were in reference to GRCh37/hg19. Significance (p < 1.13 x 10^-7^) is indicated in bold. The asterisk indicates significance of replication after Bonferroni correction for four probes (one-sided z-test). The *p*-values for MRS, Army STARRS and the combined analyses are Bonferroni-corrected for ~450K CpG sites. In stage 1, MRS and Army STARRS were combined and PRISMO was used to replicate significant findings. In stage 2, all three studies were combined. The table is organized based on significances of the DMPs in the stage 2 meta-analysis (without correction for smoking and alcohol). | | | | | | | | | | |

| **Supplemental Table S2.** SNPs within 500 bps upstream or downstream of the significant DMPs | | | | |
| --- | --- | --- | --- | --- |
| Probe (CpG) | Chr: position | SNP ID | Strand | Distance  US or DS |
| cg05656210 | 5: 141660565 | rs4998914 | F | 0 |
| cg12169700 | 7: 1923695 | rs11761270 | F | 0 |
| cg20756026 | 17: 80394529 | rs4789774 | R | 1 DS |
| Chr: chromosome, SNP: single nucleotide polymorphism, F: forward, R: reverse, US: upstream, DS: downstream | | | | |

| **Supplemental Table S3.** Differentially methylated positions (DMPs) in MRS with and without correction for main associated SNPs | | | | | | |
| --- | --- | --- | --- | --- | --- | --- |
|  | Without SNPs | | | With SNPs | | |
|  | β | SE | *P-*value | β | SE | *P-*value |
| cg05656210 | -0.37 | 0.15 | 1.6E-02 | -0.35 | 0.15 | 2.11E-02 |
| cg12169700 | -1.24 | 0.27 | 4.2E-06 | -1.29 | 0.22 | 6.46E-08 |
| cg20756026 | -0.62 | 0.21 | 3.3E-03 | -0.47 | 0.18 | 9.11E-03 |
| SE: standard error. Associated SNPs were rs7703928, rs11761270 and rs4789774, respectively. | | | | | | |

| **Supplemental Table S4.** Correlations between blood and brain methylation levels for the top CpG sites based on external data | | | | | | | | |
| --- | --- | --- | --- | --- | --- | --- | --- | --- |
|  | Brain region | | | | | | | |
| CpG | PFC | | EC | | STG | | CER | |
|  | *r* | *P-*value | *r* | *P-*value | *r* | *P-*value | *r* | *P-*value |
| cg05656210 | 0.99 | 7.54e-58 | 0.99 | 8.5E-55 | 0.99 | 2.63E-60 | 0.99 | 1.57E-57 |
| cg12169700 | 0.99 | 1.16E-62 | 0.98 | 1.32E-51 | 0.99 | 2.58E-65 | 0.93 | 1.48E-32 |
| cg20756026 | 0.99 | 1.23E-68 | 0.99 | 1.45E-67 | 0.99 | 5.32E-72 | 0.99 | 4.64E-65 |
| PFC: prefrontal cortex, EC: entorhinal cortex, STG: superior temporal gyrus, CER: cerebellum, r= Pearson correlation coefficient. Derived from http://epigenetics.essex.ac.uk/bloodbrain/. | | | | | | | | |

| **Supplemental Table S5.** Number of probes and samples removed at each stage of the quality control pipeline. | | | |
| --- | --- | --- | --- |
| **Probes QC** |  |  |  |
|  | **MRS** | **PRISMO** | **STARRS** |
| Starting N probes | 485,512 | 485,512 | 485,512 |
| Removal of > 10% missingness | 678 | 377 | 441 |
| Removal of Cross-hybridizing probes | 29,233 | 29,233 | 29,233 |
| Final N probes | 455,601 | 455,902 | 455,838 |
| **Samples QC** |  |  |  |
|  | **MRS** | **PRISMO** | **STARRS** |
| Starting N samples | 258 | 124 | 172 |
| Low sample intensity | 2 | 0 | 16 |
| Sample mismatch | 4 | 0 | 0 |
| Final N samples | 252 (126 pairs) | 124 (62 pairs) | 156 (78 pairs) |


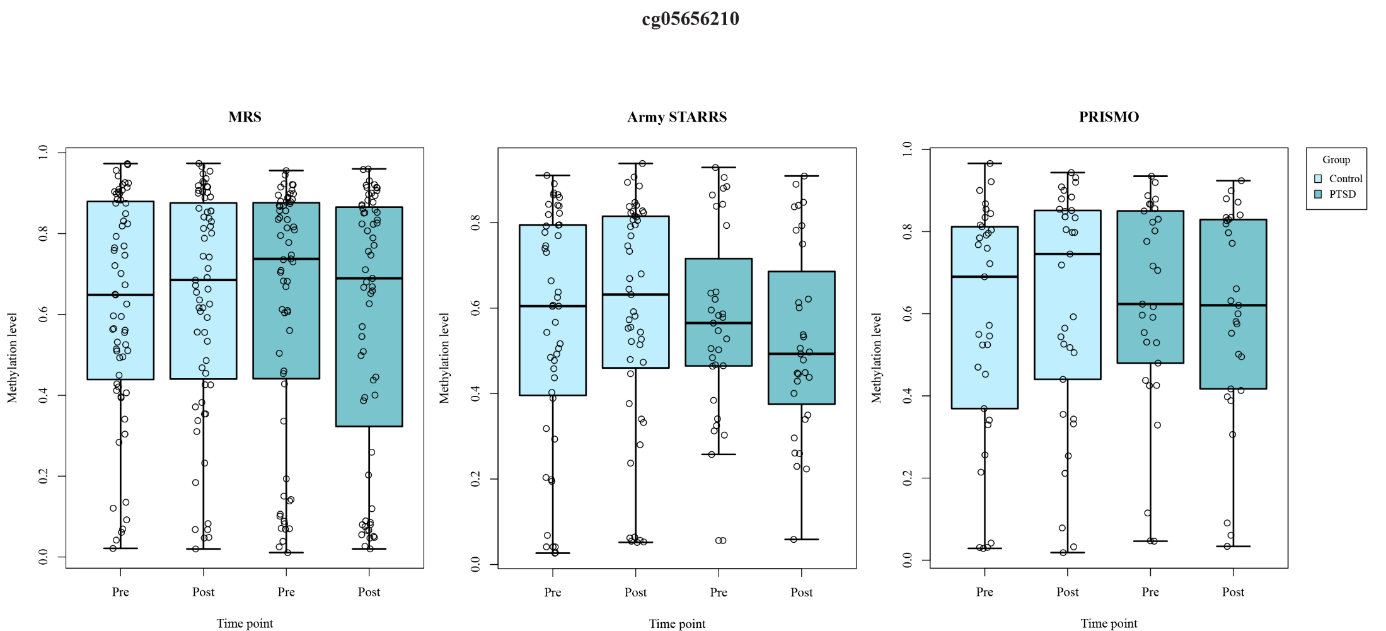


Supplemental Figure S1. Methylation values (B values) at cg05656210 for each cohort separately. Pre: pre-deployment, post: post-deployment.


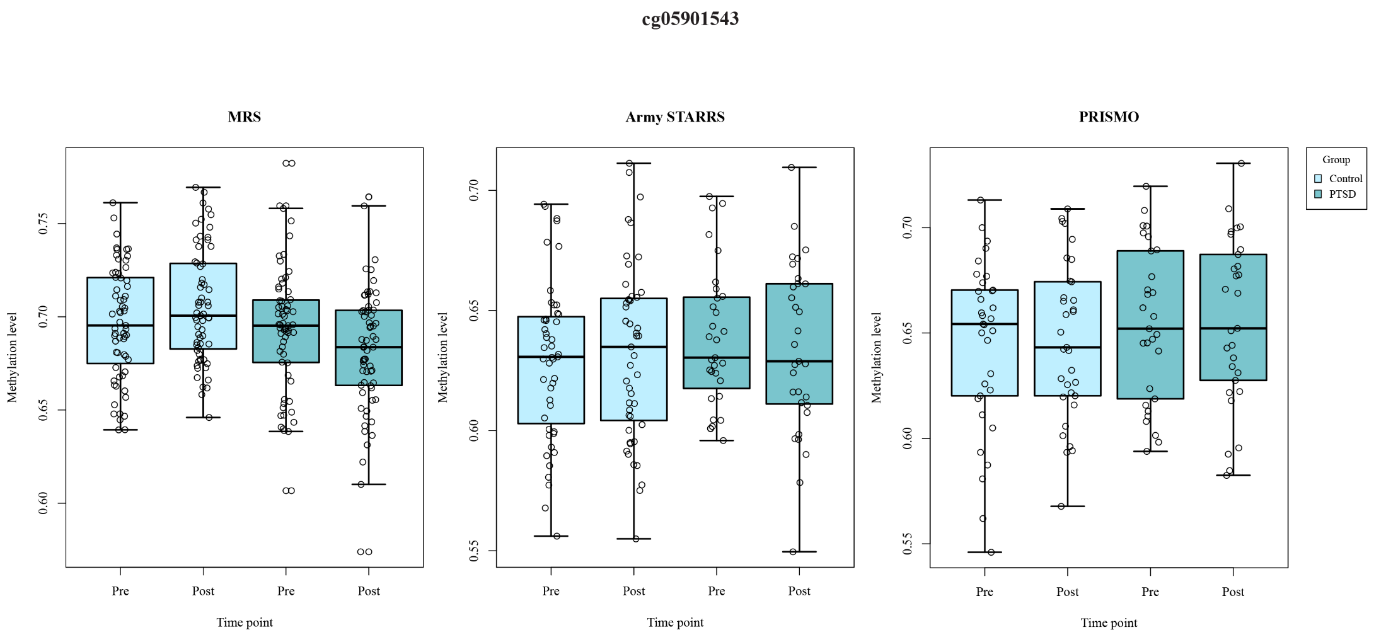


Supplemental Figure S2. Methylation values (B values) at cg05901543 for each cohort separately. Pre: pre-deployment, post: post-deployment.


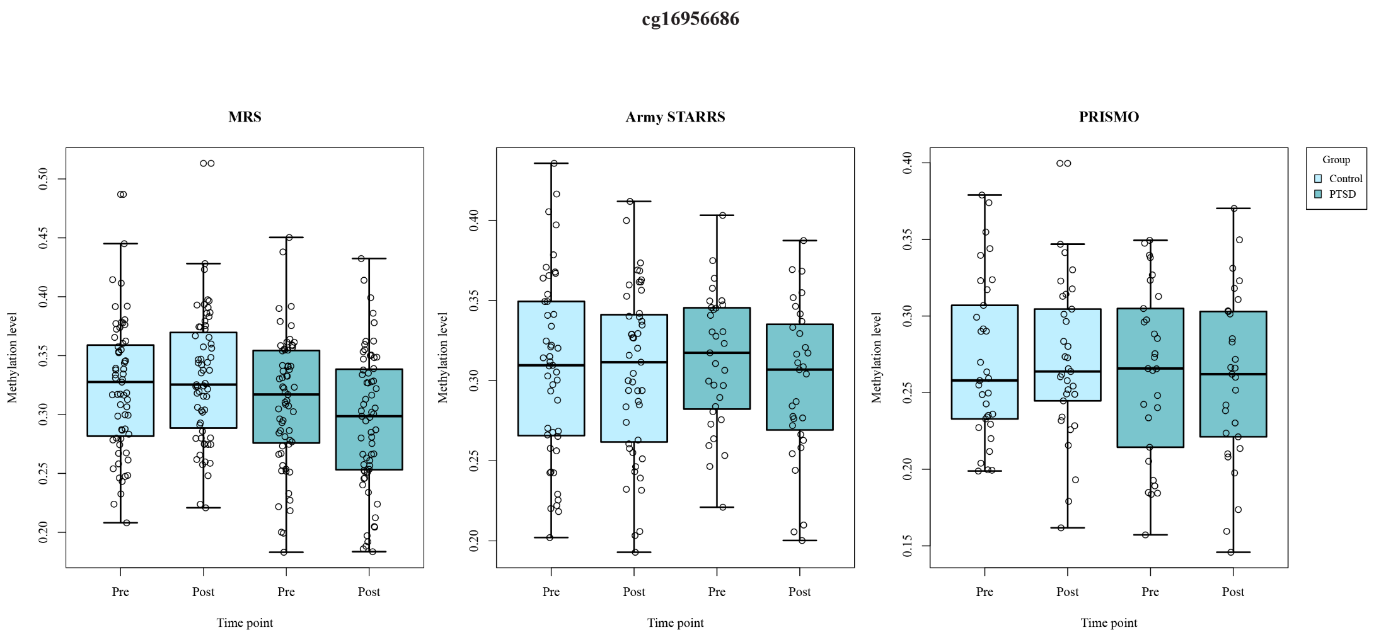


Supplemental Figure S3. Methylation values (B values) at cg16956686 for each cohort separately. Pre: pre-deployment, post: post-deployment.


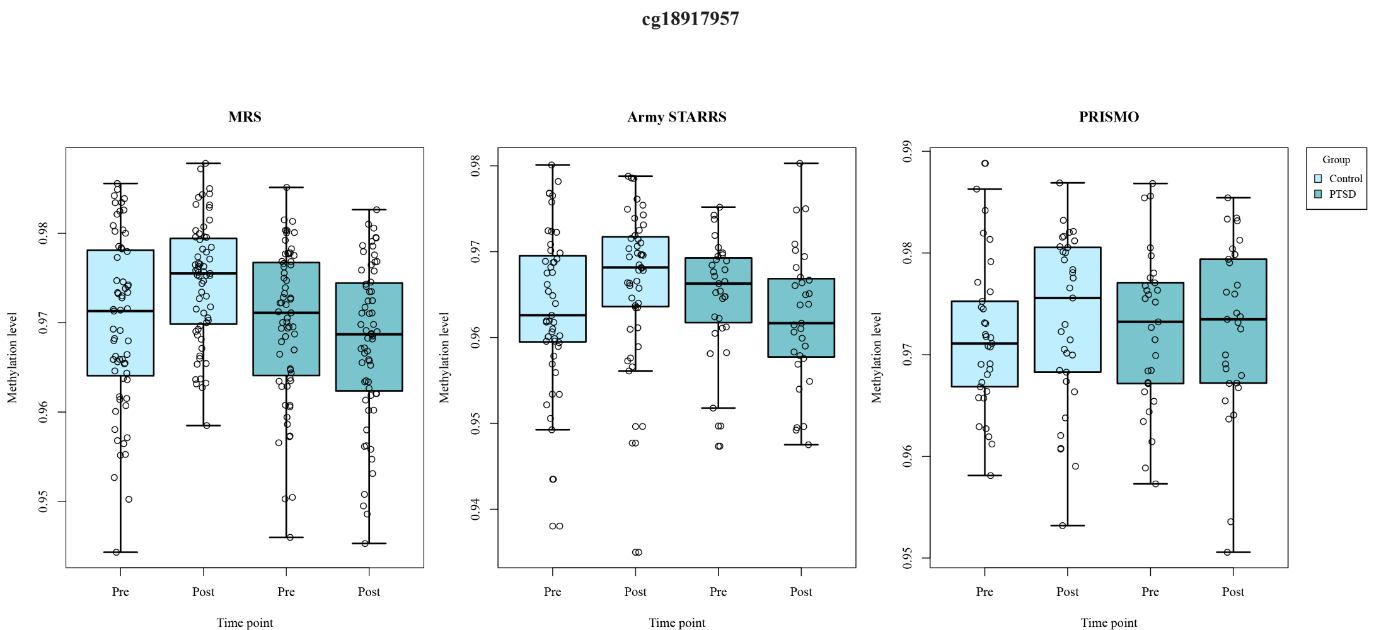


Supplemental Figure S4. Methylation values (B values) at cg18917957 for each cohort separately. Pre: pre-deployment, post: post-deployment.


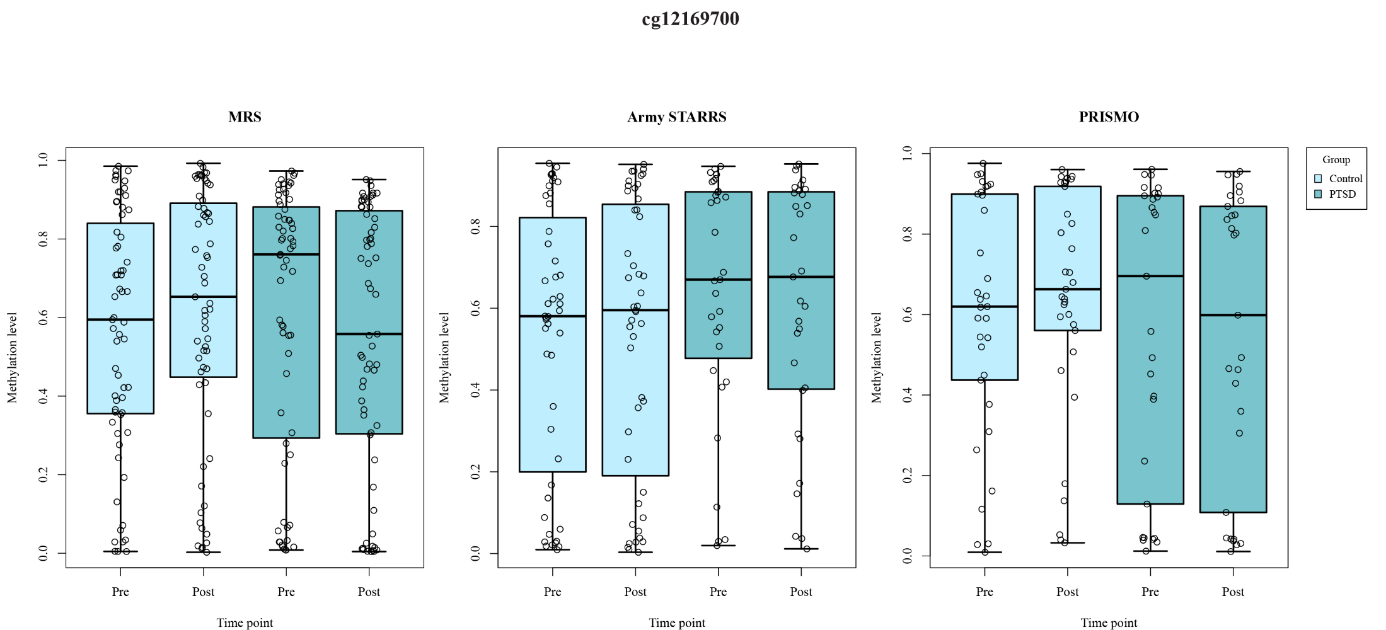


Supplemental Figure S5. Methylation values (B values) at cg12169700 for each cohort separately. Pre: pre-deployment, post: post-deployment.


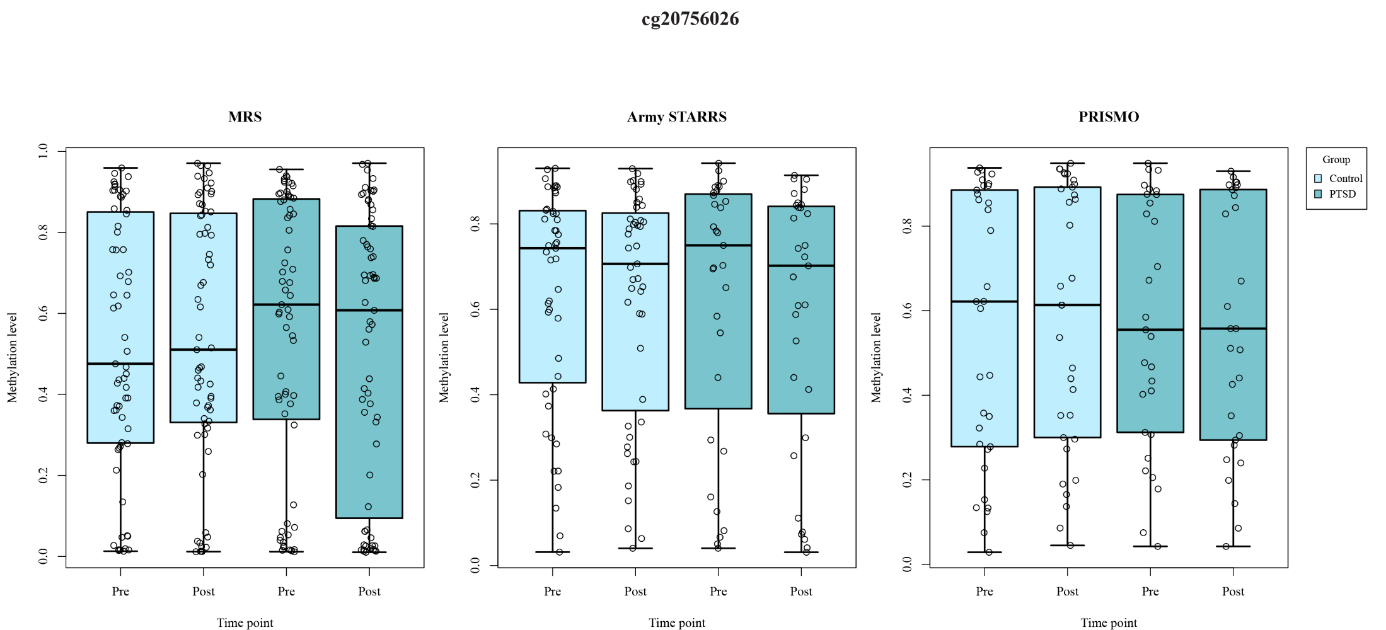


Supplemental Figure S6. Methylation values (B values) at cg20756026 for each cohort separately. Pre: pre-deployment, post: post-deployment.


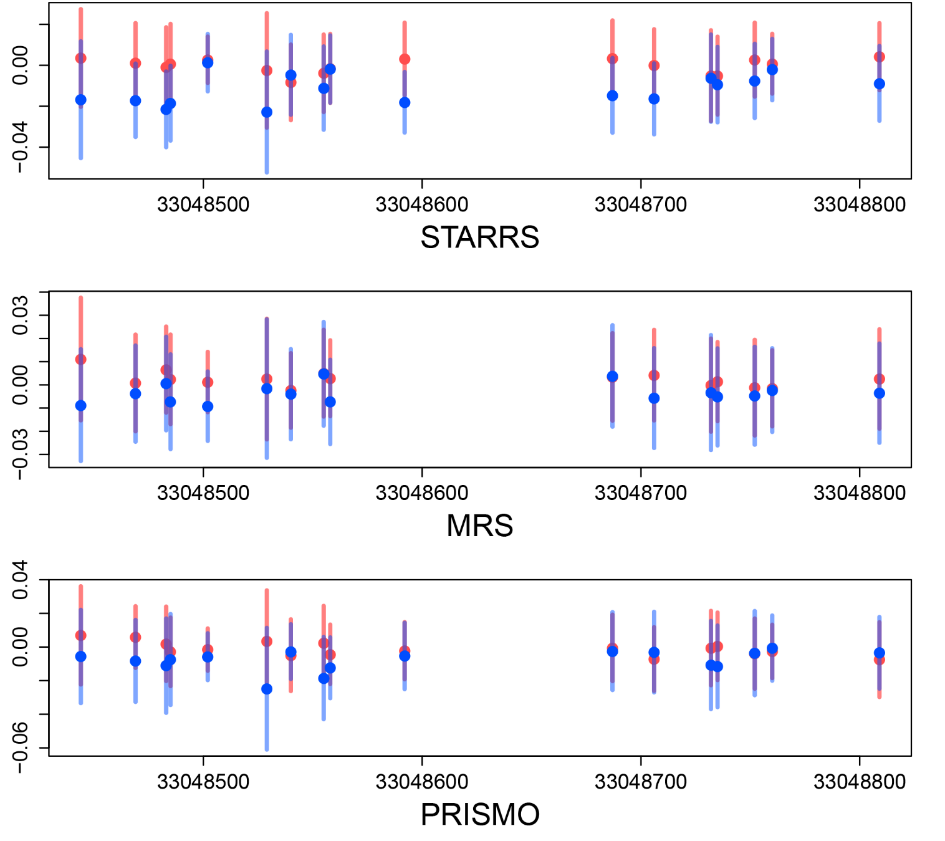


Supplemental Figure S7. Differentially methylated region at *HLA-DBP1:* 33048416-33048814. Red indicates PTSD cases, blue indicates controls.


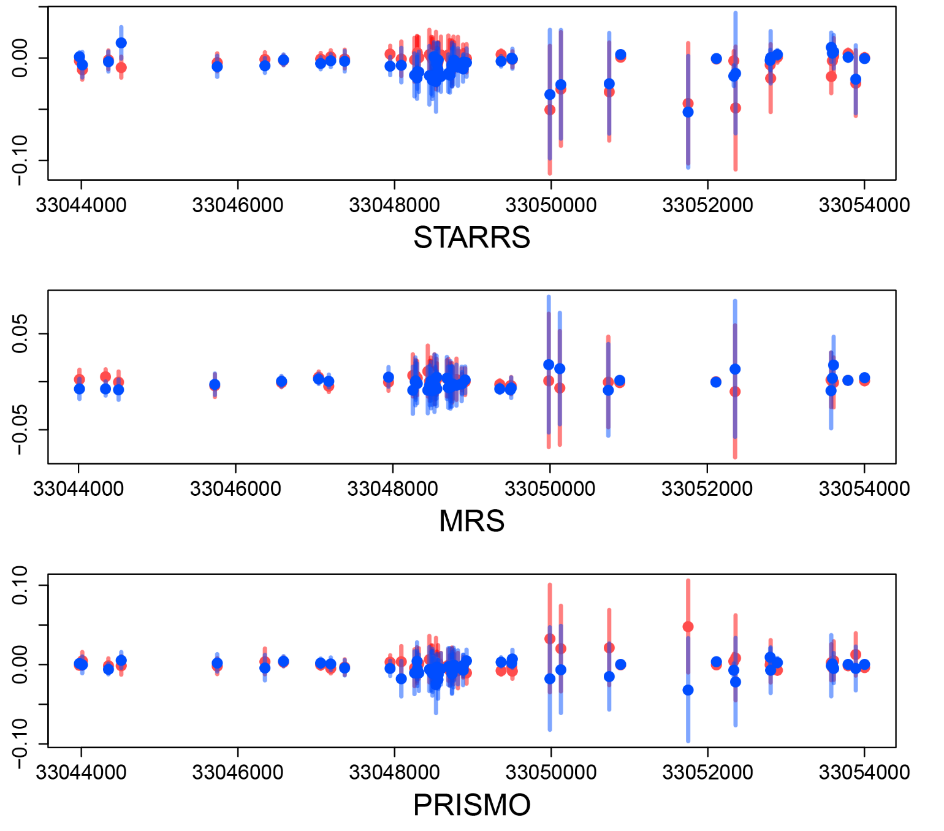


Supplemental Figure S8. Differentially methylated region at *HLA-DPB1*: 33043976-33054001. Red indicates PTSD cases, blue indicates controls.


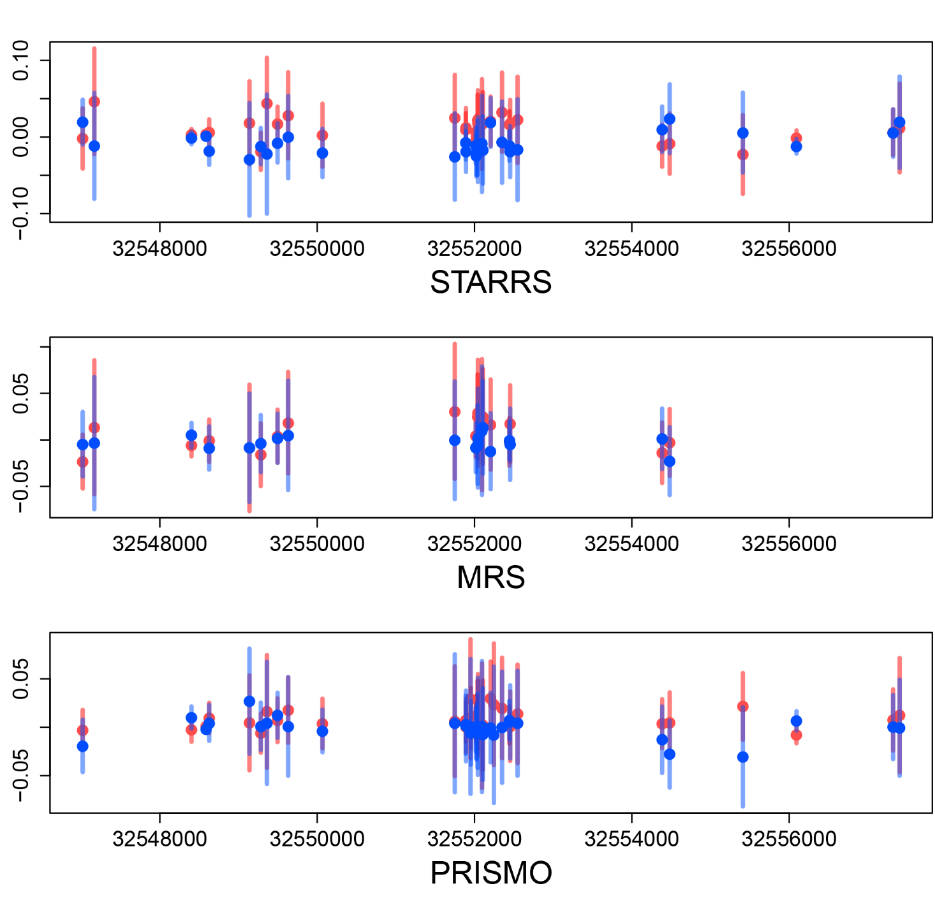


Supplemental Figure S9. Differentially methylated region at *HLA-DRB1*: 32547019-32557404. Red indicates PTSD cases, blue indicates controls.


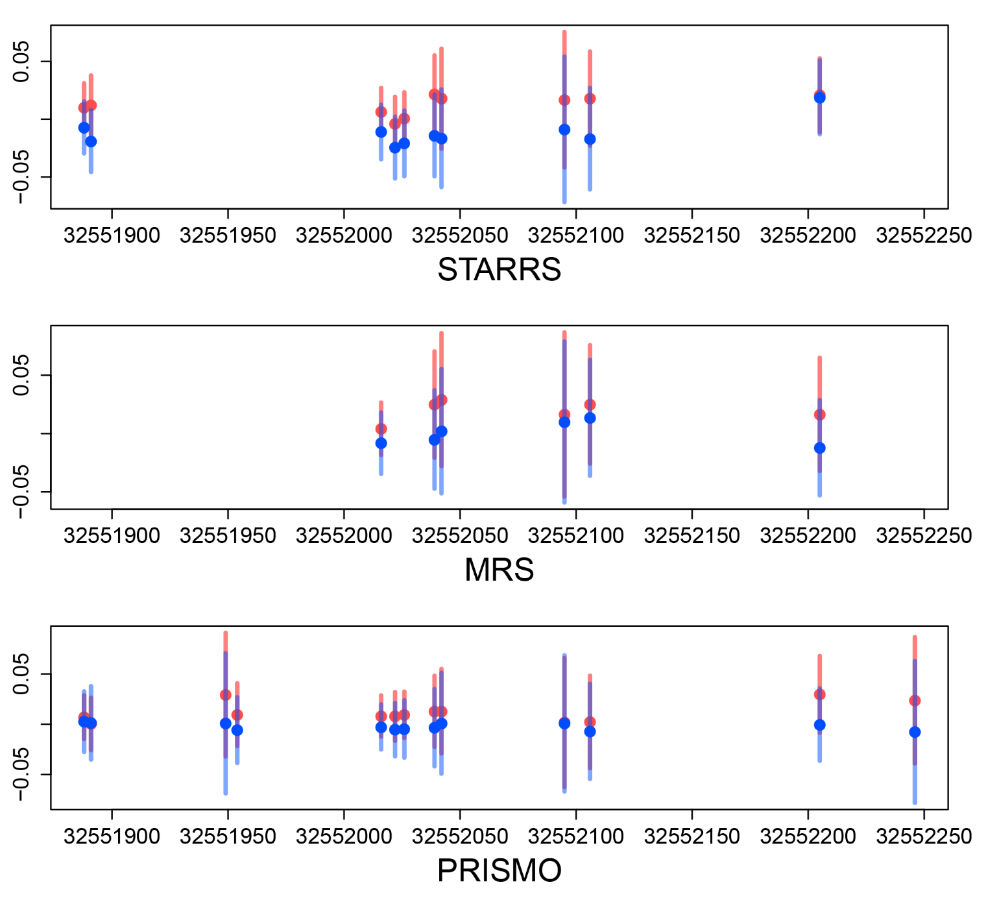


Supplemental Figure S10. Differentially methylated region at *HLA-DRB1*: 32551851-32552331. Red indicates PTSD cases, blue indicates controls.


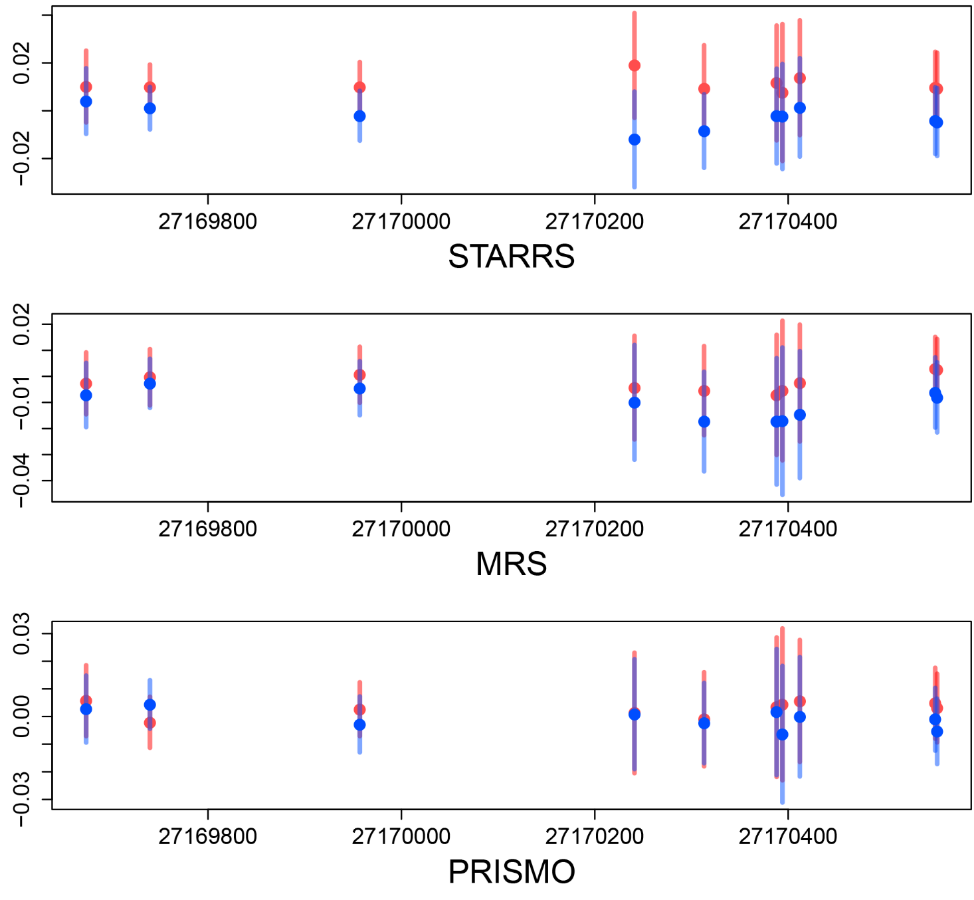


Supplemental Figure S11. Differentially methylated region at *HOXA4*: 27169572-27170638. Red indicates PTSD cases, blue indicates controls.


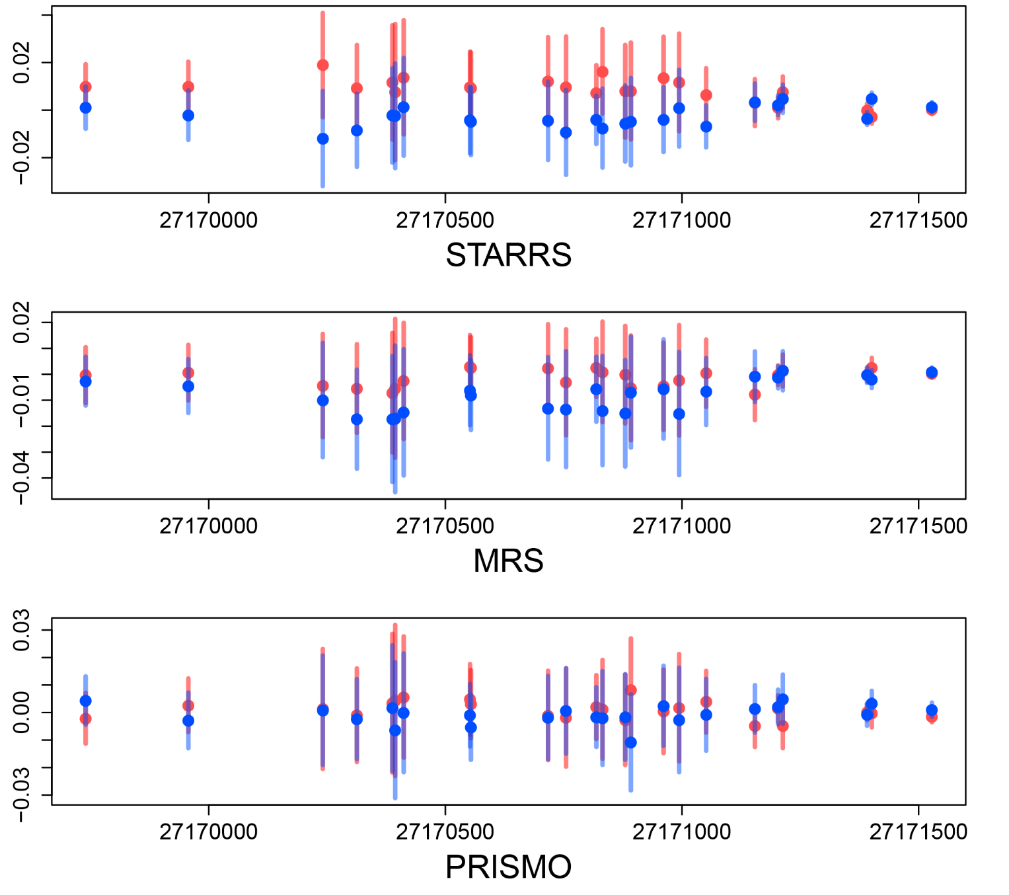


Supplemental Figure S12. Differentially methylated region at *HOXA4*: 27169740-27171528. Red indicates PTSD cases, blue indicates controls.


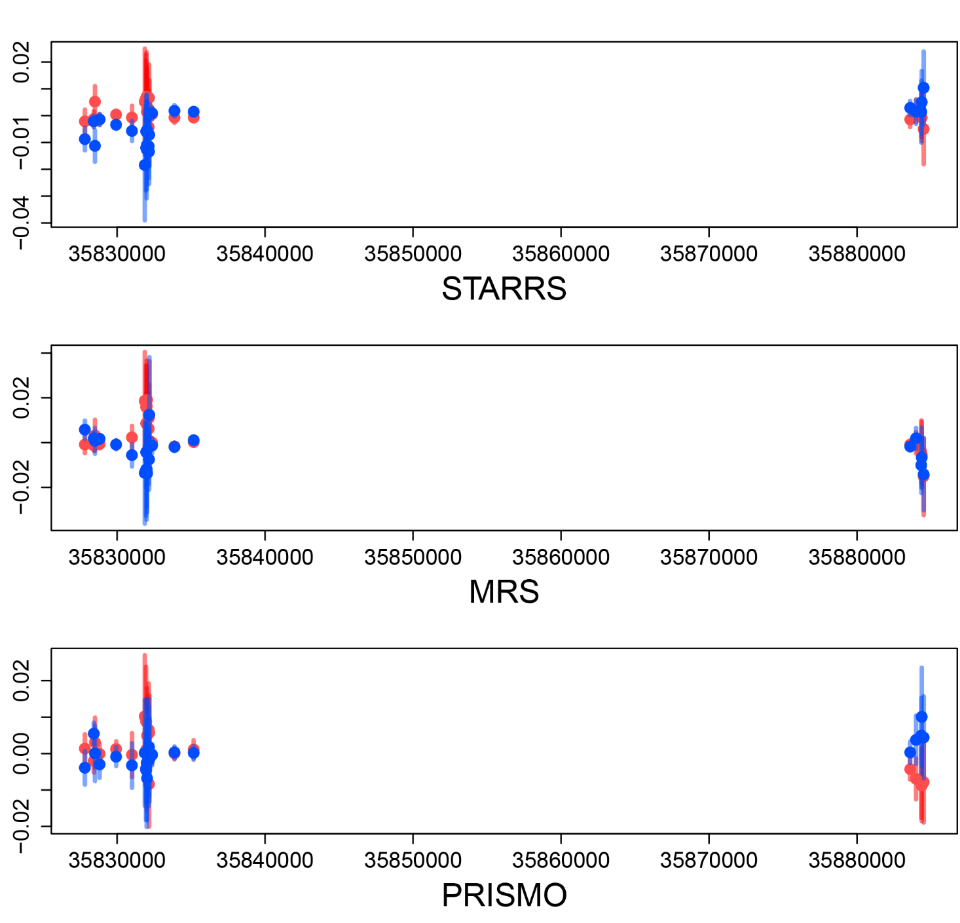


Supplemental Figure S13. Differentially methylated region at *KCNE1*: 35827824-35884508. Red indicates PTSD cases, blue indicates controls.


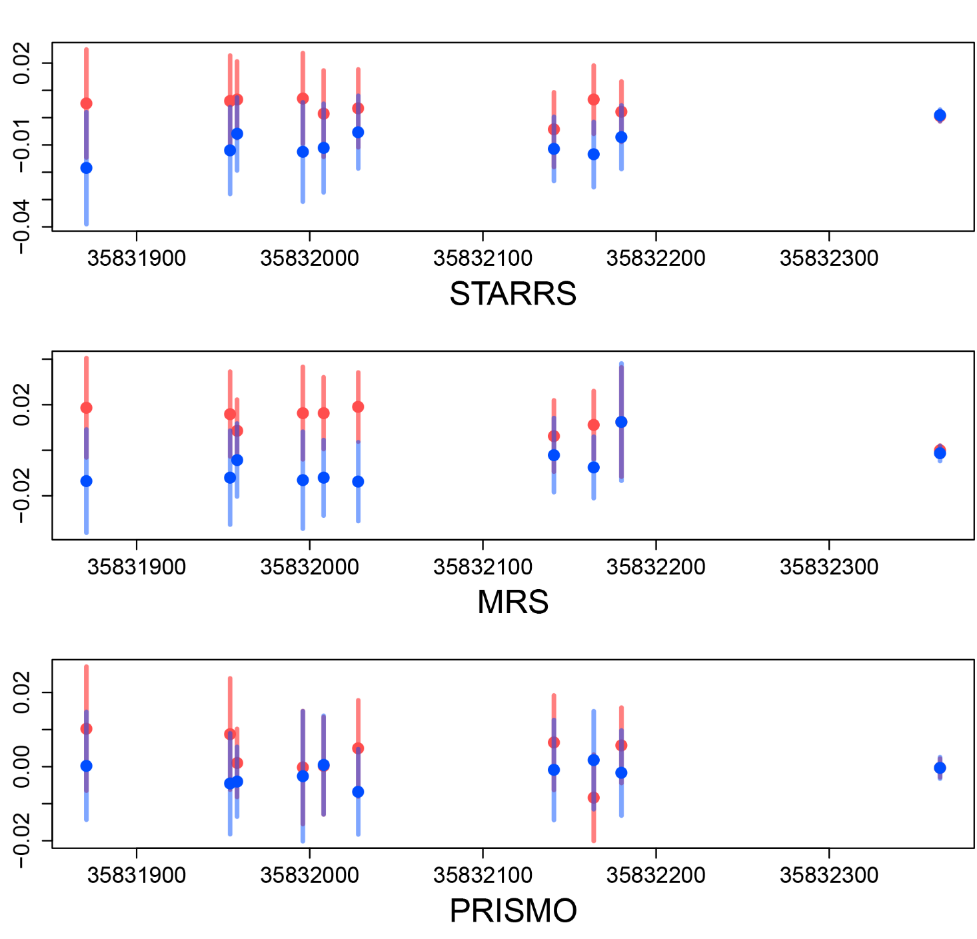


Supplemental Figure S14. Differentially methylated region at *KCNE1*: 35831697-35832365. Red indicates PTSD cases, blue indicates controls.


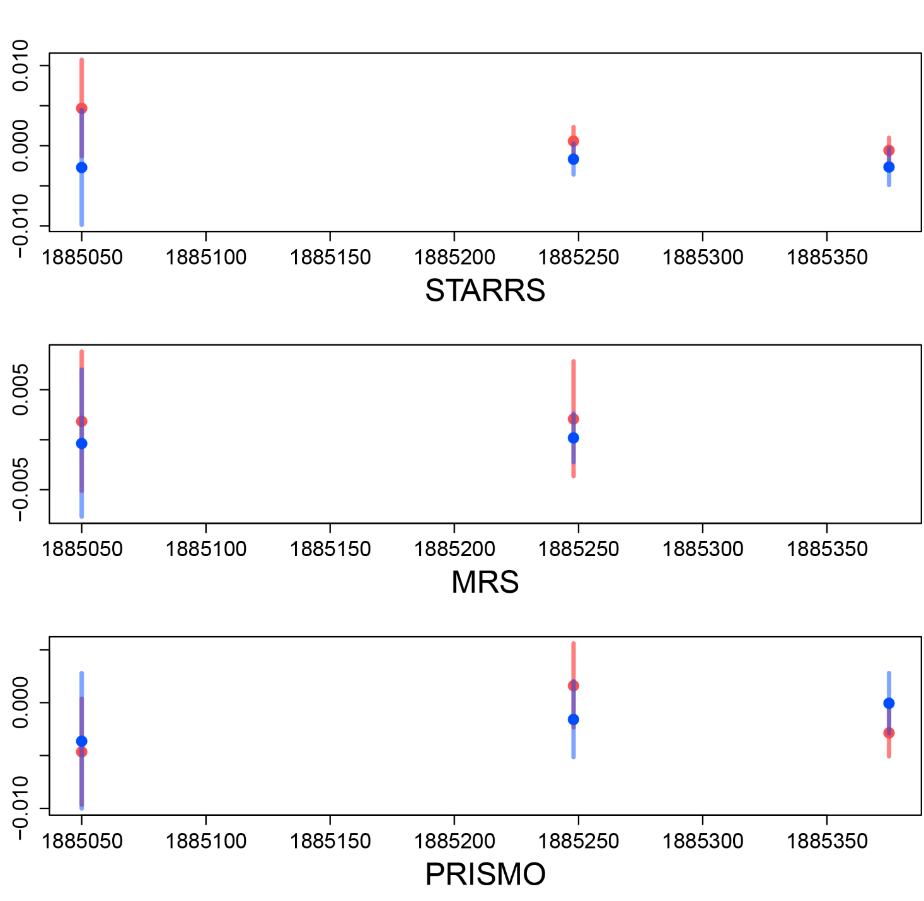


Supplemental Figure S15. Differentially methylated region at *MAD1L1*: 1885033-1885402. Red indicates PTSD cases, blue indicates controls.


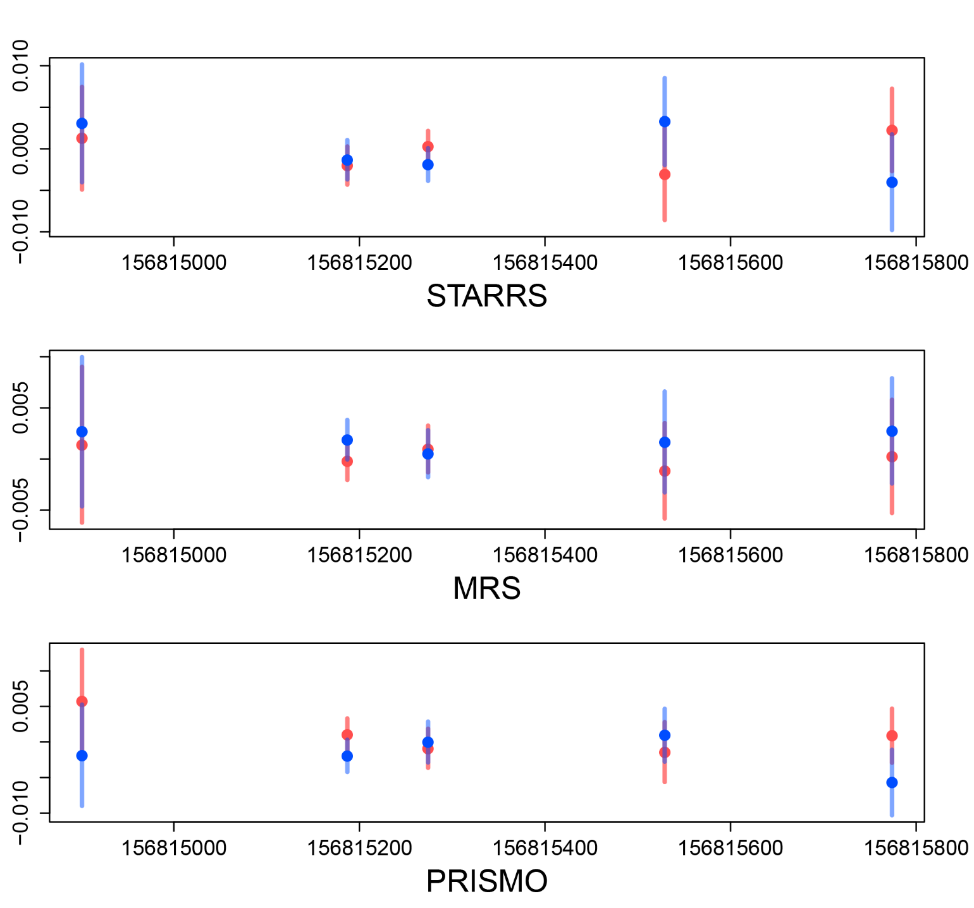


Supplemental Figure S16. Differentially methylated region at *NTRK1*: 156814881-156815792. Red indicates PTSD cases, blue indicates controls.


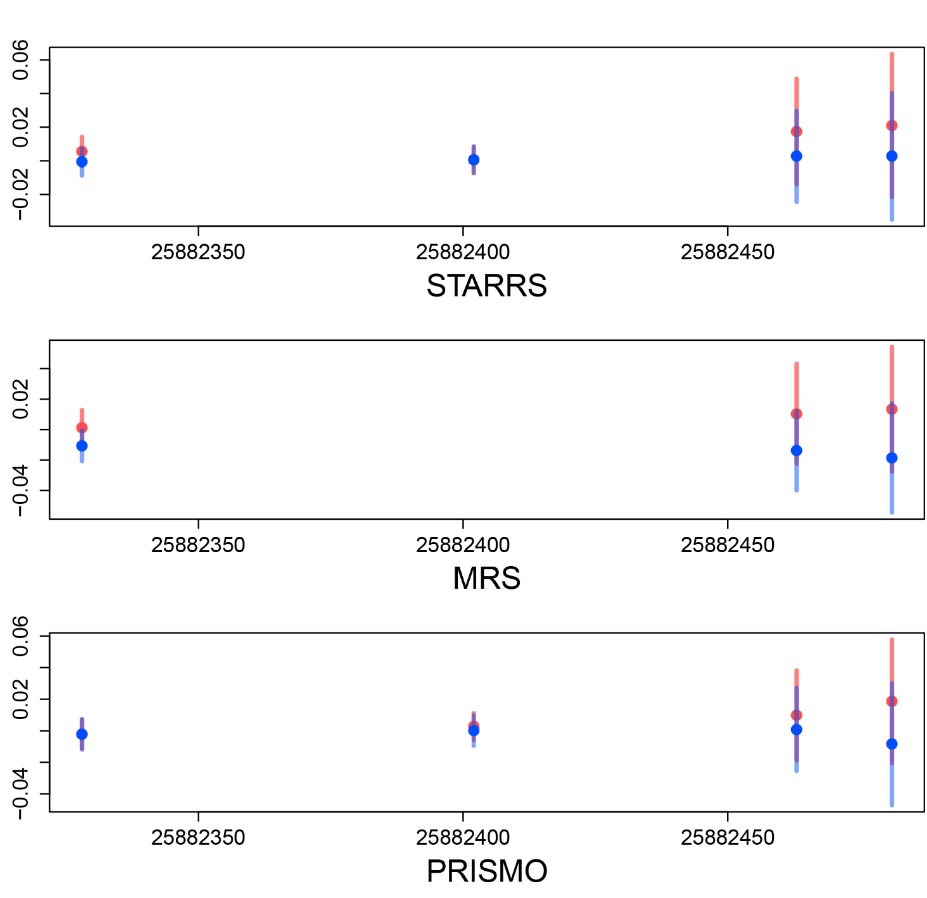


Supplemental Figure S17. Differentially methylated region at *SLC17A3*: 25882327-25882560. Red indicates PTSD cases, blue indicates controls.


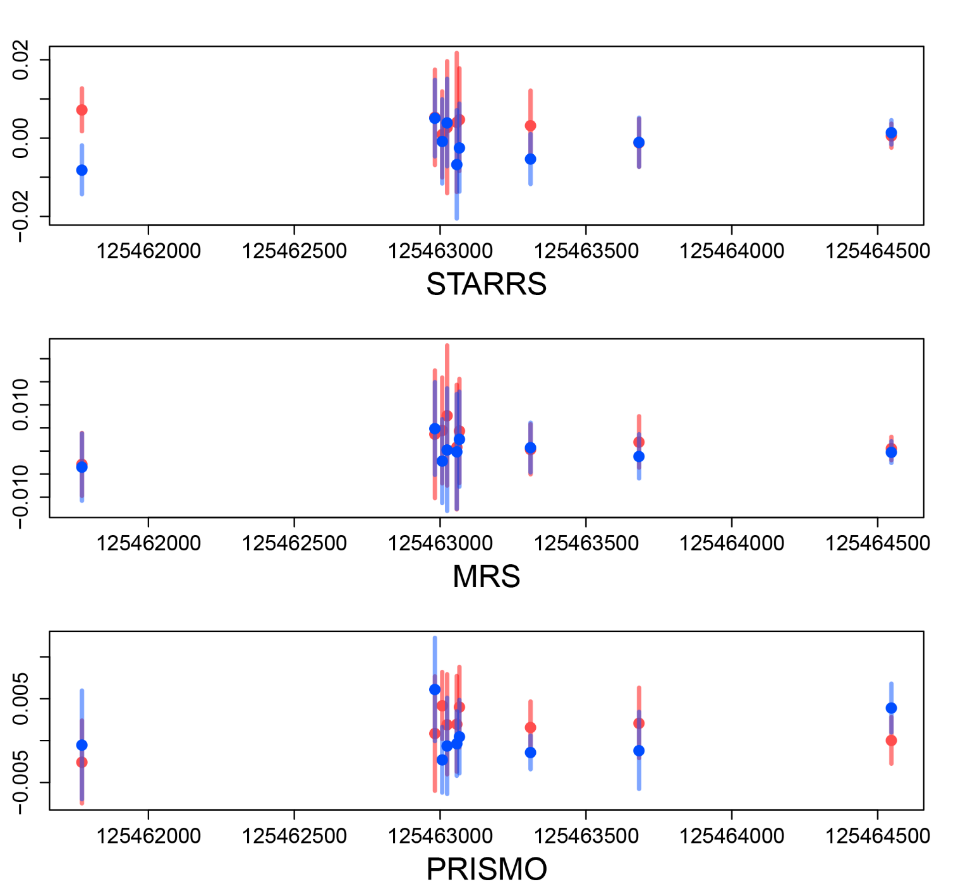


Supplemental Figure S18. Differentially methylated region at *TRMT12*: 125461772-125464547. Red indicates PTSD cases, blue indicates controls.


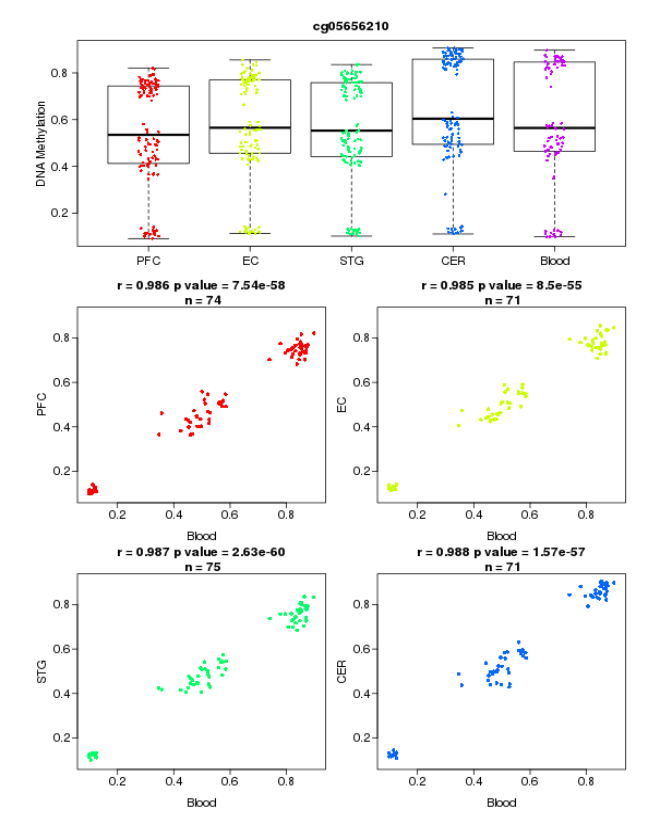


Supplemental Figure S19. Example of blood-brain correlations of methylation levels in cg05656210. PFC: prefrontal cortex, EC: entorhinal cortex, STG: superior temporal gyrus, CER: cerebellum.
